# Acute Inflammation Alters Brain Energy Metabolism in Mice and Humans: Role in Suppressed Spontaneous Activity, Impaired Cognition, and Delirium

**DOI:** 10.1523/JNEUROSCI.2876-19.2020

**Published:** 2020-07-15

**Authors:** John Kealy, Carol Murray, Eadaoin W. Griffin, Ana Belen Lopez-Rodriguez, Dáire Healy, Lucas Silva Tortorelli, John P. Lowry, Leiv Otto Watne, Colm Cunningham

**Affiliations:** ^1^School of Biochemistry and Immunology, Trinity Biomedical Sciences Institute & Trinity College Institute of Neuroscience, Trinity College Dublin, Dublin 2, Ireland; ^2^Department of Chemistry, Maynooth University, Maynooth, Ireland; ^3^Oslo Delirium Research Group, Department of Geriatric Medicine, Oslo University Hospital, Nydalen N-0424, Norway

**Keywords:** cognitive, delirium, dementia, hypoglycemia, IL-1, sepsis

## Abstract

Systemic infection triggers a spectrum of metabolic and behavioral changes, collectively termed sickness behavior, which while adaptive, can affect mood and cognition. In vulnerable individuals, acute illness can also produce profound, maladaptive, cognitive dysfunction including delirium, but our understanding of delirium pathophysiology remains limited. Here, we used bacterial lipopolysaccharide (LPS) in female C57BL/6J mice and acute hip fracture in humans to address whether disrupted energy metabolism contributes to inflammation-induced behavioral and cognitive changes. LPS (250 µg/kg) induced hypoglycemia, which was mimicked by interleukin (IL)-1β (25 µg/kg) but not prevented in IL-1RI^−/−^ mice, nor by IL-1 receptor antagonist (IL-1RA; 10 mg/kg). LPS suppression of locomotor activity correlated with blood glucose concentrations, was mitigated by exogenous glucose (2 g/kg), and was exacerbated by 2-deoxyglucose (2-DG) glycolytic inhibition, despite preventing IL-1β synthesis. Using the ME7 model of chronic neurodegeneration in female mice, to examine vulnerability of the diseased brain to acute stressors, we showed that LPS (100 µg/kg) produced acute cognitive dysfunction, selectively in those animals. These acute cognitive impairments were mimicked by insulin (11.5 IU/kg) and mitigated by glucose, demonstrating that acutely reduced glucose metabolism impairs cognition selectively in the vulnerable brain. To test whether these acute changes might predict altered carbohydrate metabolism during delirium, we assessed glycolytic metabolite levels in CSF in humans during inflammatory trauma-induced delirium. Hip fracture patients showed elevated CSF lactate and pyruvate during delirium, consistent with acutely altered brain energy metabolism. Collectively, the data suggest that disruption of energy metabolism drives behavioral and cognitive consequences of acute systemic inflammation.

**SIGNIFICANCE STATEMENT** Acute systemic inflammation alters behavior and produces disproportionate effects, such as delirium, in vulnerable individuals. Delirium has serious short and long-term sequelae but mechanisms remain unclear. Here, we show that both LPS and interleukin (IL)-1β trigger hypoglycemia, reduce CSF glucose, and suppress spontaneous activity. Exogenous glucose mitigates these outcomes. Equivalent hypoglycemia, induced by lipopolysaccharide (LPS) or insulin, was sufficient to trigger cognitive impairment selectively in animals with existing neurodegeneration and glucose also mitigated those impairments. Patient CSF from inflammatory trauma-induced delirium also shows altered brain carbohydrate metabolism. The data suggest that the degenerating brain is exquisitely sensitive to acute behavioral and cognitive consequences of disrupted energy metabolism. Thus “bioenergetic stress” drives systemic inflammation-induced dysfunction. Elucidating this may offer routes to mitigating delirium.

## Introduction

Systemic infection triggers a spectrum of metabolic and behavioral changes, termed sickness behavior, which includes fever, lethargy, hypophagia, anhedonia. Sickness behavior is an evolutionarily conserved response to illness and represents a reprioritization by the organism to conserve energy and maximize the probability of recovery (Dantzer, 2004). Systemic administration of the bacterial endotoxin lipopolysaccharide (LPS) can induce sickness behavior in humans (Schedlowski et al., 2014; Draper et al., 2018) and rodents (Teeling et al., 2007; Carlton and Demas, 2017) and despite not readily crossing the blood-brain barrier (Banks and Robinson, 2010), LPS increases central proinflammatory cytokines including interleukin (IL)-1β and tumor necrosis factor (TNF)-α (Teeling et al., 2007; Murray et al., 2011; Skelly et al., 2013), and alters local field potential (Semmler et al., 2008; Mamad et al., 2018). Peripheral inflammatory status is communicated to the brain via direct vagal signaling to the brainstem and hypothalamus; macrophage activation in the circumventricular organs lacking a patent BBB, leading to secretion of inflammatory mediators into the parenchyma; and activation of endothelial cyclooxygenases to secrete lipophilic prostaglandins directly into the parenchyma (Dantzer, 2018). Manipulation of prostaglandin-dependent mechanisms revealed neuroanatomical pathways underpinning sickness responses (Saper et al., 2012), but the molecular basis for acute LPS-induced suppression of activity is poorly understood.

Sickness behavior sometimes encompasses cognitive impairment: peripheral LPS or IL-1β administration can affect synaptic plasticity and hippocampal-dependent learning and memory (Yirmiya and Goshen, 2011), although the relative preservation of cognitive function is striking given the overt suppression of spontaneous behavior (Cunningham and Sanderson, 2008; Skelly et al., 2019). However, when inflammatory insults are severe, or occur in older age or during evolving dementia, they may induce delirium (Elie et al., 1998). Delirium is an acute onset and fluctuating syndrome characterized by inability to sustain attention, reduced awareness and perception, and profound cognitive impairment (American Psychiatric Association, 2013), affecting ∼1/5 hospital inpatients (or 1/3 for those >80 years age; Ryan et al., 2013). Delirium is associated with extended hospitalization, subsequent cognitive decline, and increased risk for dementia, but the neurobiological understanding of delirium is limited.

We have modeled delirium, using superimposition of LPS on models of neurodegeneration (Field et al., 2012; Murray et al., 2012; Cunningham and Maclullich, 2013; Lopez-Rodriguez et al., 2018) to produce acute onset, fluctuating deficits in relevant cognitive domains (Davis et al., 2015). These LPS-associated deficits are absent in normal animals but susceptibility to LPS-induced cognitive impairment increases as a function of the underlying neurodegenerative state of the brain (Griffin et al., 2013; Davis et al., 2015). LPS-induced deficits are prostaglandin dependent and can be mimicked by systemic administration of IL-1β (Griffin et al., 2013) or TNF-α (Hennessy et al., 2017) and reduced by systemic administration of IL-1 receptor antagonist (IL-1RA; Cunningham and Sanderson, 2008; Skelly et al., 2019), suggesting that IL-1β may affect cognition via a peripheral route. One possibility is that acute sickness impinges on cerebral metabolism through systemic metabolic changes; cerebral glucose uptake is reduced in a rat model of LPS-induced sepsis (Semmler et al., 2008), carbohydrate metabolism is decreased post-LPS (Irahara et al., 2018), and IL-1 has been demonstrated to induce hypoglycemia (Del Rey et al., 2006). Systemic hypoglycemia impacts on central glucose levels (Kealy et al., 2015), which in turn can affect neuronal activity and may be especially detrimental if the brain is already compromised during evolving neurodegenerative pathology.

Therefore, we hypothesized that LPS-induced disturbances in glucose metabolism would drive suppression of activity and cognitive impairment in mice. We assessed locomotor activity and working memory, while manipulating glucose metabolism with LPS, 2-deoxyglucose (2-DG), and insulin to determine effects of altered glycemic status on sickness behavior and cognitive impairments in disease-naive and ME7 mice. Finally, we analyzed carbohydrate metabolism in the cerebrospinal fluid (CSF) of inflammatory trauma (hip fracture) patients to assess brain energy metabolites in humans with inflammation-induced delirium. The findings show that altered glycemic status causes disruption of brain function in mice and that brain carbohydrate metabolism is also disrupted during delirium in patients.

## Materials and Methods

### 

#### Animals

Female c57BL/6J aged five to eight months (Harlan), mixed sex IL-1R1^−/−^ mice (six months) and mixed sex c57BL/6J aged 8–12 weeks (in-house colony) were housed at 21°C with a 12/12 h light/dark cycle (lights on 8 A.M. to 8 P.M.) with food and water available *ad libitum*. All animal experiments were in accordance with European Commission Directive 2010/63/EU and were performed following ethical approval by the TCD Animal Research Ethics Committee and licensing by the Health Products Regulatory Authority (HPRA).

#### ME7 prion model of neurodegeneration

Mice were anaesthetized using Avertin (2,2,2-tribromoethanol 50% w/v in tertiary amyl alcohol, diluted 1:40 in H_2_O; 20 ml/kg, i.p.; Sigma) and placed in a stereotaxic frame (David Kopf Instruments). A total of 1 μl of 10% w/v ME7-infected c57BL/6J or 10% w/v normal brain homogenate (NBH) in sterile PBS was infused into the dorsal hippocampus at −2.0 mm (A/P), ±1.6 mm (M/L), −1.7 mm (D/V) from Bregma as described previously (Murray et al., 2011, 2012). Mice recovered in a heated chamber, then returned to their home cage where their drinking water was supplemented with sucrose (5% w/v) and carprofen (0.05% v/v; Rimadyl, Pfizer).

#### Treatments

Mice were injected intraperitoneally with one or a combination of the following treatments using sterile saline as a vehicle: LPS from *Salmonella equine abortus* (100 or 250 µg/kg; Sigma), IL-1β (25 µg/kg; R&D Systems), IL-1RA (10 mg/kg; Kineret, Biovitrum), glucose (2 g/kg; Sigma), 2-DG (2 g/kg; Sigma), and insulin [11.5 IU/kg (400 µg/kg); Sigma]. LPS was administered 2 h before open field behavior (Except where noted as 5 hours) and 3 h before the T-maze task. Glucose was administered 30 min before any behavioral task.

#### Body temperature

Body temperature was measured in mixed sex c57BL/6J (8–12 weeks old) by subcutaneous transponders (IPTT300; BioMedic Data Systems) that were implanted under isoflurane using a custom-designed injector. The temperature was checked every 20 min with BMDS Smart Probes 7000 series Reader (BioMedic Data Systems) starting from 1 h before the saline or LPS challenge up to 240 min after the treatment.

#### Behavioral assessment

Spontaneous activity was assessed by observing locomotor activity in an open field as previously described (Murray et al., 2013). Briefly, mice were allowed to freely and individually explore an open field arena (58 × 33 × 19 cm), which was divided into squares (10 × 10 cm). Over the course of 3 min, the number of squares crossed by each mouse was counted.

Cognitive performance was assessed using an escape-from-water alternation task in a paddling T-maze as described previously (Murray et al., 2012; Skelly et al., 2019). Briefly, this working memory task involves two runs per trial. On the first run, mice only have one of the T-maze arms available to them, which has an exit at the end of it to escape from the shallow water. On the second run, mice are given a choice between the two arms with the exit now in the opposite arm to the first run. Mice were trained (two blocks/day, five trials/block, two runs/trial) until they performed with at ≥80% success. They were then pharmacologically challenged and tested on the same day of the challenge. There were three blocks of five trials postchallenge [corresponding to 3–5 (+3), 5–7 (+5), and 7–9 (+7) h postchallenge] and two blocks for insulin, due to its rapid action on blood glucose [corresponding to 40–160 min (+1 h) and 160–300 min (+3 h) postchallenge]. All mice underwent recovery testing (two blocks of five trials) on the following day.

#### Blood glucose measurements

For serial blood measurements, mice were placed in a plastic restrainer, the tail vein was dilated using warm water and lanced using a 30-G needle. Glucose was measured using a veterinary glucometer (AlphaTRAK 2, Zoetis), which assesses glucose concentration based on oxidation by flavin adenine dinucleotide (FAD) glucose dehydrogenase. This enzyme preferentially accepts glucose as substrate but shows 2-DG oxidation at 37% of the rate of glucose oxidation. However, at a dose 0.0003 mol 2-DG/25-g mouse, this 2-DG is insufficient to affect blood glucose (∼10 mm) determination. Terminal blood glucose measurements were made following sodium pentobarbital overdose and incising the right ventricle, immediately before transcardial perfusion. Blood glucose readings were higher on the veterinary glucometer compared with a clinical glucometer (data not shown), but basal levels were broadly in line with other studies (Del Rey et al., 2006, 2016; Chakera et al., 2018; Tooke et al., 2019).

#### CSF sampling and analysis

In mice, CSF was collected under terminal anesthesia. Mice were placed in a stereotaxic frame and the cisterna magna accessed by lowering the incisor bar on the animal's head to angle it downwards at 45° from horizontal. Using a small volume insulin syringe (BD Micro - Fine + 0.3 ml Insulin Syringe Demi), a freehand puncture was performed slowly to avoid brain stem damage and blood contamination. Approximately 5 μl was collected in 0.5-ml microcentrifuge tubes.

#### Hip fracture patient cohort

Hip fracture is a frequent occurrence in frail, elderly populations. Delirium occurs with high prevalence in these patients (Marcantonio, 2017), and since these patients, in many centers, receive spinal anesthesia for hip fracture repair surgery, this offers an opportunity for CSF collection allowing assessment of the impact of this acute inflammatory trauma on CSF markers of brain energy metabolism in older individuals. CSF was collected from hip fracture patients acutely admitted to Oslo University Hospital after informed consent from the patient and/or proxy (if patients were unable to consent due to cognitive impairment), as approved by the Regional Committee for Medical and Health Research Ethics (South-East Norway; REK 2009/450). The presence of delirium was assessed in all participants using the Confusion Assessment Method (CAM; Inouye et al., 1990) based on a 10- to 30-min interview with participants and information from relatives, nurses, and hospital records. One geriatrician and one old age psychiatrist independently evaluated whether participants met the ICD-10 criteria for dementia before the fracture, based on all available data, as explained earlier (Watne et al., 2014b). CSF was collected in propylene tubes at the onset of spinal anesthesia. Samples were centrifuged, aliquoted and stored at –80°C, as previously described (Watne et al., 2014a).

Samples were defrosted and transferred to CMA Microvials (CMA Microdialysis AB). In mice, there were two occasions where sample volumes were too small (<3 μl) for analysis. In these cases, two samples from the same treatment groups were pooled (see Fig. 2*H*,*I*). All other mouse CSF measurements were made on samples taken from individual mice. Glucose, lactate, and pyruvate (the latter in humans only) concentrations were determined using a CMA600 Microdialysis Analyzer (CMA Microdialysis AB). The CMA600 uses a colorimetric analysis technique to detect these analytes. Using glucose as an example, the CMA reagent works by metabolism of glucose by glucose oxidase to form its gluconic acid and hydrogen peroxide. The hydrogen peroxide then reacts with phenol to produce a color change and this is detected by a photometric sensor. While 2-DG (used as a treatment in some mice that undergo this analysis) can react with glucose oxidase, its reactivity is much lower than that of glucose (V_max_ around 50 vs glucose 1150 M s^−1^; Gibson et al., 1964). In practice, 2-DG treatment has no significant effect on CSF glucose readings based on our data (see Fig. 2*K*). Basal levels of CSF glucose were in the range expected from other studies (Horn and Klein, 2010; Nakamura et al., 2017; Tang et al., 2017), as was the case for CSF lactate (Horn and Klein, 2010). The lower limits of detection were: glucose (0.1 mmol/l), lactate (0.1 mmol/l), and pyruvate (4 μmol/l). Since the ability to detect pyruvate is an indication of its concentration, non-detected samples probably represent very low concentrations of pyruvate. However, samples where no value was returned by the assay were assigned the value of 2 μmol/l (i.e., 50% of the lowest value that we did detect) as a conservative measure.

#### Experimental design and statistical analyses

Statistical analysis was performed in GraphPad Prism 5 and IBM SPSS version 25. Pairs of groups were measured using *t* tests, and all multiple comparisons were made using ANOVAs, paired and repeated measure variants were used as appropriate. Full statistical analyses and experimental numbers are included in figure legends. Where data were found to violate the assumptions of *t* tests (using a combination of visual inspection, Kolmogorov–Smirnov and the Shapiro–Wilk normality tests), the non-parametric Mann–Whitney *U* test was used instead. The group numbers, statistical tests used, and test values are all summarized in Table 1.

**Table 1.** Summary of experimental design and statistical analyses used in this study

Figure	Group (*n*)	Test used	Statistical values	*p* value	*Post hoc* tests
1*B*	*c57BL6* mice: Saline at 2 h (8) Saline at 4 h (6) LPS at 2 h (6) LPS at 4 h (6)	2 × 2 between groups ANOVA	Treatment_(saline;LPS)_ *F*_(1,22)_ = 36.71 Time_(2,4 h)_ *F*_(1,22)_ = 5.916 Treatment × time *F*_(1,22)_ = 5.916	<0.0001 0.0236 0.0236	Bonferroni LPS vs saline at 2 h: *t* = 6.215; *p* < 0.001 LPS vs saline at 4 h: *t* = 2.483; *p* < 0.05
1*C*	*c57BL6* mice: Saline (6) LPS (7)	2 × 5 repeated measures ANOVA	Treatment_(saline;LPS)_ *F*_(1,44)_ = 24.10 Time_(0,3,5,7,24 h)_ *F*_(4,44)_ = 5.054 Treatment × time *F*_(4,44)_ = 3.019	0.0005 0.0019 0.0276	Bonferroni LPS vs saline at 5 h: *t* = 3.362; *p* < 0.01 LPS vs saline at 7 h: *t* = 4.575; *p* < 0.001
1*D*	*c57BL6* mice: Saline+saline (6) IL-1β+saline (7) IL-1β+IL-1RA_10 µg_ (7)	3 × 6 repeated measures ANOVA	Treatment_(saline;IL-1β,IL-1RA10μg)_ *F*_(2,85)_ = 3.843 Time_(0,1,3,5,7,24 h)_ *F*_(5,85)_ = 19.39 Treatment × time *F*_(10,85)_ = 3.442	0.0420 <0.0001 0.0008	Bonferroni IL-1β+Sal vs Sal+Sal 1 h: *t* = 3.566; *p* < 0.01 IL-1β+Sal vs Sal+Sal 3 h: *t* = 3.409; *p* < 0.01 IL-1β+Sal v IL-1β+IL-1RA 3 h: *t* = 3.013; *p* < 0.05
1*E*	LPS/IL-1 in WT, IL-1RI^−/−^ mice c57BL6+saline (6) c57BL6+LPS (6) c57BL6+ IL-1β (5) IL-1R1^−/−^+saline (6) IL-1R1^−/−^+LPS (6) IL-1R1^−/−^+IL-1β (6)	2 × 3 between groups ANOVA	Genotype_(c57;1L-1R1)_ *F*_(1,29)_ = 2.938 Treatment_(saline;LPS;IL-1β)_ *F*_(2,29)_ = 21.81 Genotype × treatment *F*_(2,29)_ = 2.782	0.0972 <0.0001 0.0785	Bonferroni Between genotypes: IL-1β – c57BL6 v IL-1R1^−/−^ *t* = 2.836; *p* < 0.05 Within genotypes: c57BL6 – LPS vs Sal *t* = 4.287; *p* < 0.001 c57BL6 – IL-1β vs Sal *t* = 2.465; *p* < 0.05 IL-1R1^−/−^ – LPS vs Sal *t* = 4.763; *p* < 0.001
1*F*	LPS in WT, IL-1R1^−/−^ c57BL6+LPS (7) IL-1R1^−/−^+LPS (5)	2 × 2 repeated measures ANOVA	Genotype_(c57;1L-1R1)_ *F*_(1,40)_ = 5.673 Time_(0,1,3,5,7,24 h)_ *F*_(4,40)_ = 22.78 Genotype × time *F*_(4,40)_ = 1.460	0.0385 <0.0001 0.2324	Bonferroni No significant differences between genotypes at any time.
1*G*	IL-1RA-treated^-^ mice: Saline+saline (12) LPS+saline (12) LPS+IL-1RA_10_ (12)	3 × 5 repeated measures ANOVA	Treatment_(Sal;IL-1β,IL-1RA10μg)_ *F*_(2,132)_ = 16.18 Time_(0,2,4,6,24 h)_ *F*_(4,132)_ = 39.08 Treatment × time *F*_(8,132)_ = 3.502	<0.0001 <0.0001 0.0011	Bonferroni Between treatments: LPS+Sal vs Sal+Sal 2 h *t* = 3.449; *p* < 0.01 LPS+Sal vs Sal+Sal 4 h *t* = 5.160; *p* < 0.001 LPS+Sal vs Sal+Sal 6 h *t* = 4.319; *p* < 0.001 LPS+IL-1RA vs Sal+Sal 4 h: *t* = 4.551; *p* < 0.001 LPS+IL-1RA_10_ vs Sal+Sal 6 h: *t* = 3.817; *p* < 0.001
2*A*	*c57BL6* mice: LPS (8) Saline (8)	6 × 2 repeated measures ANOVA	Treatment_(saline;LPS)_ *F*_(1,14)_ = 9.74 Time_(−60,40,120,240,420 min, 24 h)_ *F*_(3,40)_ = 11.09	0.0075 <0.0001	Bonferroni LPS vs Sal at 4 h *t* = 3.576; *p* < 0.050 LPS vs Sal at 7 h *t* = 4.456; *p* < 0.010
2*B*	*c57BL6* mice: LPS (5) Saline (6)	14 × 2 repeated measures ANOVA	Time_(−60−240 min,every 20 min)_ *F*_(3,24)_ = 5.166 Treatment: *F*_(1,9)_ = 0.6231	0.0082 0.4502	Bonferroni No significant differences between treatments at any time
2*C*	*c57BL6* mice: LPS (8) Saline (12)	3 × 2 repeated measures ANOVA	Treatment_(saline;LPS)_ *F*_(1,18)_ = 6.43 Time_(0,5,24 h)_ *F*_(1,22)_ = 29.46	0.0207 <0,0001	Bonferroni LPS vs Sal at 5 h *t* = 6.854; *p* < 0.0001
2*D*	*c57BL6* mice: LPS (8) Saline (12)	3 × 2 repeated measures ANOVA	Treatment_(saline;LPS)_ *F*_(1,15)_ = 10.11 Time_(0,5,24 h)_ *F*_(1,20)_ = 9.458	0,0062 0,0035	Bonferroni LPS vs Sal at 5 h *t* = 3.122; *p* < 0.05
3*A*	*c57BL6* mice: Saline (14) LPS (14)	Linear regression	Saline: slope = −3.512 ± 7.51*; r*^2^ = 0.01790 LPS: slope = 9.121 ± 2.439*; r*^2^ = 0.4824	0.6484 0.0020	
3*B*	*c57BL6* mice: (movement) Saline (5) LPS (5)	2 × 3 repeated measures ANOVA	Treatment_(saline;LPS)_ *F*_(1,16)_ = 0.7815 Movement_(spontaneous;prompted;total)_ *F*_(2,16)_ = 43.20 Treatment × movement *F*_(2,16)_ = 11.98	0.4025 <0.0001 0.0007	Bonferroni Between treatments: Spontaneous: LPS vs Sal *t* = 3.457; *p* < 0.01 Within treatments: LPS (Spont. vs prompt) *t* = 7.060; *p* < 0.001 LPS (Spont. vs total) *t* = 8.015; *p* < 0.001
3*D*	*c57BL6* mice: Saline+saline (7) Saline+glucose (7) LPS+saline (8) LPS+glucose (7)	2 × 2 between groups ANOVA	LPS treatment_(saline;LPS)_ *F*_(1,25)_ = 29.88 Glucose treatment_(saline;glucose)_ *F*_(1,25)_ = 0.8106 LPS × glucose *F*_(1,25)_ = 0.2352	<0.0001 0.3765 0.6319	Bonferroni Sal+Sal vs LPS+Sal *t* = 3.580; *p* < 0.01 Sal+glucose vs LPS+glucose *t* = 4.142; *p* < 0.001
3*D*	*c57BL6* mice: LPS+saline (8) LPS+Sal+2-DG (5)	Unpaired *t* test, two-tailed	*t*_(11)_ = 2.498	0.0296	
3*E*	*c57BL6* mice: Saline+saline (7) Saline+glucose (7) LPS+saline (8) LPS+glucose (9)	2 × 2 between groups ANOVA	LPS treatment_(saline;LPS)_ *F*_(1,27)_ = 13.39 Glucose treatment_(saline;glucose)_ *F*_(1,27)_ = 1.494 LPS × glucose *F*_(1,27)_ = 10.48	0.0011 0.2322 0.0032	Bonferroni Sal+Sal vs LPS+Sal *t* = 4.813; *p* < 0.001 LPS+Sal vs LPS+glucose *t* = 3.315; *p* < 0.01
3*E*	*c57BL6* mice: LPS+saline (8) LPS+Sal+2-DG (7)	Unpaired *t* test, two-tailed	*t*_(13)_ = 5.766	<0.0001	
3*F*	*c57BL6* mice: Saline+saline (7) Saline+glucose (7) LPS+saline (8) LPS+glucose (9)	2 × 2 between groups ANOVA	LPS treatment_(Sal;LPS)_ *F*_(1,27)_ = 60.00 Glucose treatment_(saline;glucose)_ *F*_(1,27)_ = 6.721 LPS × glucose *F*_(1,27)_ = 0.7495	<0.0001 0.0152 0.3943	Bonferroni Sal+Sal vs LPS+Sal *t* = 6.010; *p* < 0.001 Sal+Gluc. vs LPS+Gluc. *t* = 4.931; *p* < 0.001 LPS+Sal vs LPS+glucose *t* = 2.571; *p* < 0.05
3*F*	*c57BL6* mice: LPS+saline (8) LPS+Sal+2-DG (7)	Unpaired *t* test, two-tailed	*t*_(13)_ = 2.567	0.0234	
3*G*	*c57BL6* mice: LPS+saline (6) LPS+glucose (6)	2 × 6 repeated measures ANOVA	Treatment_(LPS+saline;LPS+glucose)_ *F*_(1,50)_ = 6.681 Time_(−1,2,4,6,18,24 h)_ *F*_(5,50)_ = 32.95 Treatment × time *F*_(5,50)_ = 9.029	0.0272 <0.0001 <0.0001	Bonferroni Between treatments: LPS+Sal vs LPS+glucose:2 h *t* = 6.784; *p* < 0.001 Within treatments: LPS+glucose, –1 vs 2 h *t* = 4.730; *p* < 0.001
3*H*	*c57BL6* mice: Saline+saline (7) Saline+glucose (5) LPS+saline (4) LPS+glucose (8)	2 × 2 between groups ANOVA	LPS treatment_(saline; LPS)_ *F*_(1,22)_ = 39.85 Glucose treatment_(saline; glucose)_ *F*_(1,22)_ = 14.57 LPS × glucose *F*_(1,22)_ = 0.007560	<0.0001 0.0009 0.9315	Bonferroni Sal+Sal vs LPS+Sal *t* = 4.235; *p* < 0.001 Sal+glucose vs LPS+glucose *t* = 4.720; *p* < 0.001 Sal+Sal vs Sal+glucose *t* = 2.795; *p* < 0.05 LPS+Sal vs LPS+glucose *t* = 2.606; *p* < 0.05
3*H*	*c57BL6* mice: LPS+saline (4) LPS+Sal+2-DG (4)	Unpaired *t* test, two-tailed	*t*_(7)_ = 3.367	0.0120	
3*I*	*c57BL6* mice: Saline+saline (7) Saline+glucose (5) LPS+saline (4) LPS+glucose (7)	2 × 2 between groups ANOVA	LPS treatment_(saline;LPS)_ *F*_(1,21)_ = 2.165 Glucose treatment_(saline;glucose)_ *F*_(1,21)_ = 0.008456 LPS × glucose *F*_(1,21)_ = 0.4037	0.1560 0.9276 0.5321	Bonferroni No analyses done: no main effects in ANOVA
3*J*	*c57BL6* mice: Saline (13) 2-DG (16)	Unpaired *t* test, two-tailed	*t*_(27)_ = 1.894	0.069	
3*K*	*c57BL6* mice: Saline (13) 2-DG (16)	Mann–Whitney *U* test, two-tailed	*U* = 74.0	0.1951	
4*A*	*c57BL6* mice: NBH+saline (20) NBH+LPS (21) ME7+saline (9) ME7+LPS (26)	4 × 5 repeated measures ANOVA	Treatment_(NBH+saline;NBH+LPS;ME7+saline; ME7+LPS)_ *F*_(3,288)_ = 19.08 Time_(−24,3,5,7,24 h)_ *F*_(4,288)_ = 1.146 Treatment × time *F*_(12,288)_ = 4.999	<0.0001 0.3351 <0.0001	Bonferroni Between treatments: NBH+LPS vs ME7+LPS 3 h: *t* = 2.608; *p* < 0.05 ME7+Sal vs ME7+LPS: 5 h: *t* = 4.933; *p* < 0.001 ME7+Sal vs ME7+LPS 7 h: *t* = 3.510; *p* < 0.01
4*B*	*c57BL6* mice: NBH+saline (4) NBH+insulin_90_ (5) NBH+insulin_180_ (4) ME7+saline (4) ME7+insulin_90_ (4) ME7+insulin_180_ (5)	2 × 3 between groups ANOVA	Disease_(NBH; ME7)_ *F*_(1,20)_ = 3.649 Treatment_(saline;insulin90min;insulin180min)_ *F*_(2,20)_ = 17.11 Disease × treatment *F*_(2,20)_ = 0.04292	0.0705 <0.0001 0.9581	Bonferroni Between treatments: NBH+Sal vs NBH+insulin_90_ *t* = 3.829; *p* < 0.01 NBH+Sal vs NBH+insulin_180_ *t* = 3.447; *p* < 0.01 ME7+Sal vs ME7+insulin_90_ *t* = 3.756; *p* < 0.01 ME7+Sal vs ME7+insulin_180_ *t* = 3.357; *p* < 0.01
4*C*	*c57BL6* mice: NBH+saline (6) NBH+insulin_90_ (5) NBH+insulin_180_ (4) ME7+saline (10) ME7+insulin_90_ (4) ME7+insulin_180_ (5)	2 × 3 between groups ANOVA	Disease_(NBH;ME7)_ *F*_(1,28)_ = 0.09033 Treatment_(saline;insulin90min;insulin180min)_ *F*_(2,28)_ = 22.86 Disease × treatment *F*_(2,28)_ = 0.2126	0.7660 <0.0001 0.8098	Bonferroni Between Tx: NBH+Sal vs NBH+insulin_90_ *t* = 4.691; *p* < 0.001 ME7+Sal vs ME7+insulin_90_ *t* = 4.772; *p* < 0.001
4*D*	*c57BL6* mice: NBH+saline (7) NBH+insulin (9) ME7+saline (21) ME7+insulin (12)	4 × 4 repeated measures ANOVA	Treatment_(NBH+Sal;ME7+ Sal;NBH+insulin; ME7+insulin)_ *F*_(3,135)_ = 7.418 Time_(−24,1,3,24 h)_ *F*_(3,135)_ = 6.986 Treatment × time *F*_(9,135)_ = 3.050	0.0004 0.0002 0.0024	Bonferroni Between Tx: ME7+Sal v ME7+insulin:1 h *t* = 3.235; *p* < 0.01 ME7+Sal v ME7+insulin: 3 h *t* = 3.500; *p* < 0.01
5*A*	*c57BL6* mice: NBH+saline (10) NBH+LPS (7) ME7+saline (11) ME7+LPS (7)	2 × 2 between groups ANOVA	Disease_(NBH;ME7)_ *F*_(1,31)_ = 0.07914 Treatment_(saline;LPS)_ *F*_(1,31)_ = 118.3 Disease × treatment *F*_(1,31)_ = 0.7479	0.7803 <0.0001 0.3938	Bonferroni NBH+Sal vs NBH+LPS *t* = 7.011; *p* < 0.001 ME7+saline vs ME7+LPS *t* = 8.381; *p* < 0.001
5*B*	*c57BL6* mice: NBH+saline (5) NBH+LPS (7) ME7+saline (7) ME7+LPS (7)	2 × 2 between groups ANOVA	Disease_(NBH;ME7)_ *F*_(1,22)_ = 6.665 Treatment_(saline;LPS)_ *F*_(1,22)_ = 146.5 Disease × treatment *F*_(1,22)_ = 3.605	0.0170 <0.0001 0.0708	Bonferroni NBH+Sal vs NBH+LPS *t* = 6.910; *p* < 0.001 ME7+Sal vs ME7+LPS *t* = 10.39; *p* < 0.001 NBH+Sal vs ME7+Sal *t* = 3.033; *p* < 0.05
5*C*	*c57BL6* mice: NBH+saline (5) NBH+LPS (7) ME7+saline (7) ME7+LPS (7)	2 × 2 between groups ANOVA	Disease_(NBH;ME7)_ *F*_(1,22)_ = 0.0001099 Treatment_(saline;LPS)_ *F*_(1,22)_ = 0.2568 Disease × treatment *F*_(1,22)_ = 0.003127	0.9917 0.6173 0.9559	Bonferroni No *post hoc*s performed
5*D*	ME7-inoculated *c57BL6* mice: Sal+Sal (12) Sal+glucose (13) LPS+Sal (20) LPS+glucose (19)	2 × 5 repeated measures ANOVA	Treatment_(Sal+Sal;Sal+glucose; LPS+Sal;LPS+glucose)_ *F*_(3,240)_ = 13.57 Time_(−24,3,5,7,24 h)_ *F*_(4,240)_ = 3.865 Treatment × time *F*_(12,240)_ = 3.740	<0.0001 0.0046 <0.0001	Bonferroni Between groups: 3 h: LPS+Sal vs Sal+Sal *t* = 4.230; *p* < 0.001 5 h: LPS+Sal vs Sal+Sal *t* = 6.426; *p* < 0.001 5 h: LPS+glucose vs LPS+sal *t* = 3.543; *p* < 0.01 5 h: Sal+glucose vs LPS+glucose *t* = 2.826; *p* < 0.05
6*A*	Hip fracture patients: Glucose No delirium (32) Delirium (39)	Mann–Whitney *U* test, two-tailed	*U* = 606.5	0.8442	
6*B*	Hip fracture pts: Lactate No delirium (32) Delirium (40)	Mann–Whitney *U* test, a priori one-tailed	*U* = 442.5	0.0128	
6*C*	Hip fracture pts: Lactate No dementia (55) Dementia (59)	Mann–Whitney *U* test, two-tailed	*U* = 1438	0.2954	
6*D*	Hip fracture pts: Pyruvate No delirium (32) Delirium (40)	Mann–Whitney *U* test, two-tailed	*U* = 514.5	0.0494	
6*E*	Hip fracture pts: Pyruvate No delirium (32) Delirium (39)	Fisher's exact test, two-sided		0.0306	
6*F*	Hip fracture pts: Lactate/glucose ratio (LGR) No delirium (32) Delirium (39)	Mann–Whitney *U* test, two-tailed	*U* = 399.5	0.0048	
6*G*	*c57BL6* mice (LGR) NBH+saline (5) NBH+LPS (7) ME7+saline (7) ME7+LPS (7)	2 × 2 between groups ANOVA	Disease_(NBH;ME7)_ *F*_(1,22)_ = 2.313 Treatment_(saline;LPS)_ *F*_(1,22)_ = 44.58 Disease × treatment *F*_(1,22)_ = 0.007732	0.1425 <0.0001 0.9307	Bonferroni NBH+Saline vs NBH+LPS *t* = 4.580; *p* < 0.001 ME7+saline vs ME7+LPS *t* = 4.887; *p* < 0.001

## Results

### LPS and IL-1β both robustly reduce systemic glucose concentrations

**Figure 1.**
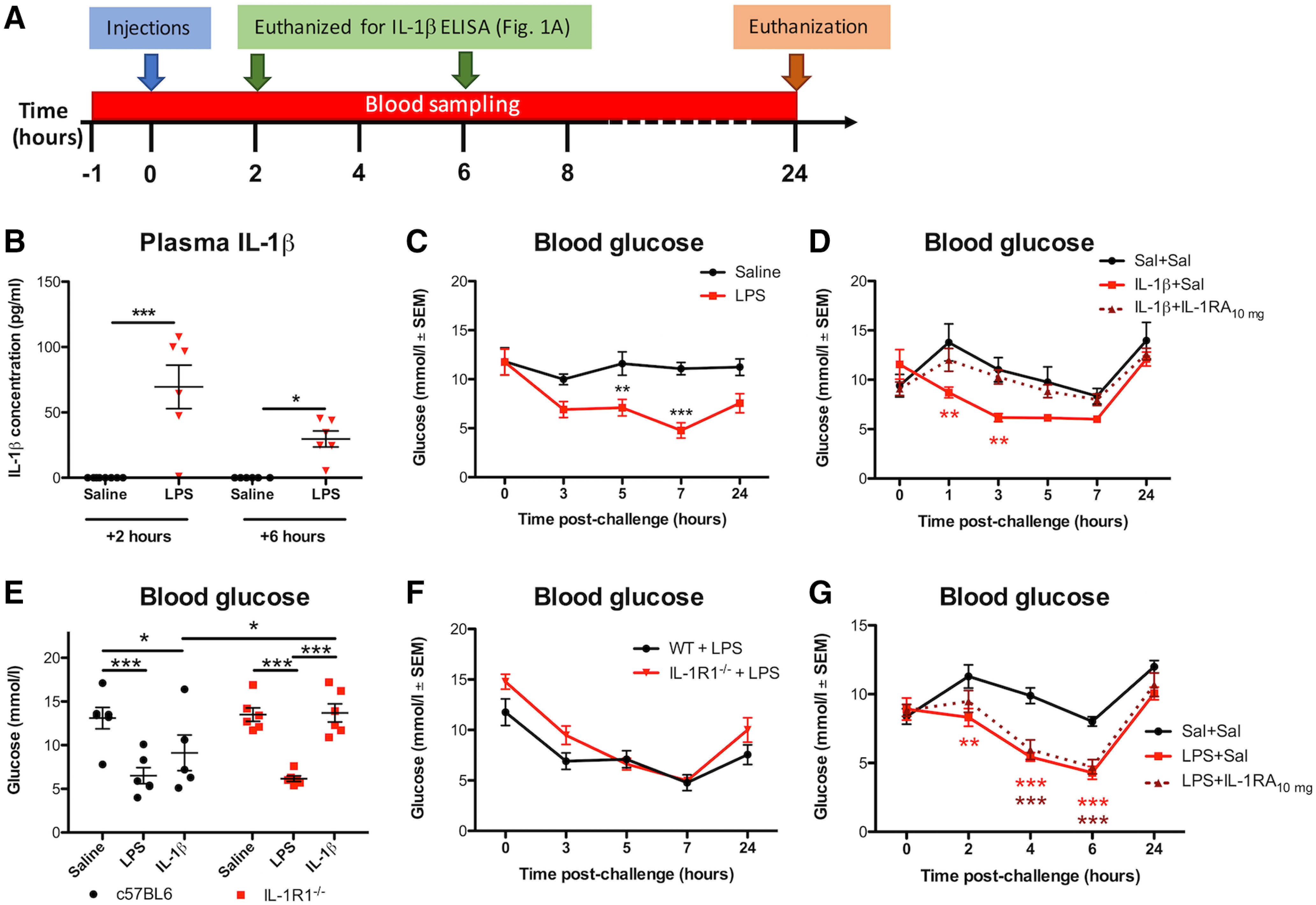
LPS and IL-1β significantly lower blood glucose concentrations. ***A***, Timeline for treatments and sampling times. Blood sampling was from tail vein, aside from the 24 hours (h) time point where glucose levels were measured from right atrial blood before transcardial perfusion. In one cohort, mice were euthanized at 2 and 6 h post-LPS challenge to collect plasma for the IL-1β Enzyme linked immunoabsorbent assay (ELISA). ***B***, LPS treatment (250 μg/kg, i.p.) significantly increased plasma IL-1β (*F*_(1,22)_ = 36.71; *p* < 0.0001; *n* = 8 for saline/2 h group; *n* = 6 for other groups). ***C***, LPS treatment (*n* = 7) significantly reduced glucose levels over 24 h compared with saline controls (0.9%, i.p.; *n* = 6); main effect of treatment (*F*_(1,44)_ = 24.10; *p* = 0.0005). ***D***, IL-1β (25 μg/kg, i.p.; *n* = 7) reduced systemic glucose and IL-1β's effect can be blocked using IL-1RA (10 mg/kg, i.p.; *n* = 7). Main effect of treatment (*F*_(2,85)_ = 3.843; *p* = 0.0420); **significantly lower glucose levels in IL-1β+saline-treated mice compared with controls (*n* = 6) at 1 and 3 h postchallenge. ***E***, c57BL/6J mice, both LPS (*n* = 6) and IL-1β (*n* = 5) significantly reduced blood glucose 4 h postchallenge versus saline controls (*n* = 6), while in IL-1R1^−/−^ mice, LPS (*n* = 6) but not IL-1 (*n* = 6) significantly reduced blood glucose versus controls (*n* = 6). Significant pairwise comparisons by Bonferroni *post hoc* test after a main effect of treatment (*F*_(2,29)_ = 21.81; *p* < 0.0001) are annotated by * (*p* < 0.05) and *** (*p* < 0.001). ***F***, Time course of changes in blood glucose in IL-1R1^−/−^ (*n* = 5) and c57BL/6J mice (*n* = 7; significant effect of genotype, *F*_(1,40)_ = 5.673; *p* = 0.0385, but no pairwise differences at any time point). ***G***, IL-1RA (10 mg/kg, *n* = 12) administered immediately after LPS treatment modestly attenuated LPS-induced reductions in glucose (*F*_(2,132)_ = 16.18; *p* < 0.0001), but this was a transient effect (*F*_(4,132)_ = 39.08; *p* < 0.001). There was a significant interaction of treatment and time (*F*_(8,132)_ = 3.502; *p* = 0.0011), and *post hoc* tests indicated that LPS+saline-treated mice (*n* = 12) had significantly lower blood glucose levels versus saline (*n* = 12) at 2, 4 and 6 h postchallenge, while LPS+IL-1RA (*n* = 12) did not significantly decrease glucose levels compared with controls until 4 h. All annotated Bonferroni *post hoc* tests were performed after significant main effects or interactions in ANOVA analysis: *p < 0.05, **p < 0.01, ***p < 0.001. All data are expressed as mean ± standard error of the mean (SEM).

The effects of systemic infection on spontaneous activity have largely been attributed to changes in cytokine signaling (Dantzer, 2018). IL-1 is reported to act at the endothelium or at forebrain targets to mediate LPS-induced suppression of motivated behaviors (Liu et al., 2019), but precisely how suppression of exploratory activity occurs is unclear. Here, we examined LPS-induced suppression of activity (see Fig. 1*A*) and confirmed that LPS (250 μg/kg, i.p.) significantly increased plasma IL-1β levels at 2 and 6 h postchallenge in c57BL/6J mice compared with saline-treated controls (Fig. 1*B*) and also reduced blood glucose levels by >50% by 7 h postchallenge (Fig. 1*C*). Blood glucose was decreased as early as 3 h following LPS and had not fully returned to baseline levels by 24 h. This reduction is not explained by suppression of feeding since blood glucose declines rapidly here (Fig. 1*B*,*E*) but only begins to decrease after 6–12 h of fasting in healthy c57BL/6J mice (Champy et al., 2004).

We investigated the contribution of IL-1β signaling to LPS-induced hypoglycemia. IL-1β (25 μg/kg, i.p.) reduced blood glucose, with a more rapid induction and earlier nadir than LPS (Fig. 1*D*). IL-1β-induced reductions in glucose were completely blocked by IL-1RA (10 mg/kg i.p.). Therefore, IL-1β is sufficient to reduce systemic glucose levels.

To test whether IL-1β is necessary for LPS-induced hypoglycemia, we administered LPS (250 μg/kg, i.p.) and IL-1β (25 μg/kg, i.p.) to IL-1 receptor-1 knock-out (IL-1R1^−/−^) mice and c57BL/6J wild-type (WT) controls. Blood glucose measurements were taken 4 h postchallenge, to ensure robustly decreased glucose (Fig. 1*C*,*D*). LPS and IL-1β again reduced blood glucose in WT mice. Although IL-1β-induced hypoglycemia was prevented in IL-1R1^−/−^ mice, LPS-induced reductions in glucose were statistically indistinguishable from those in WTs (Fig. 1*E*). Moreover, the time course of LPS-induced glucose reduction was highly overlapping in WT and IL-1RI^−/−^ mice (Fig. 1*F*). IL-1β antagonism with IL-1RA has been reported to attenuate LPS-induced hypoglycemia (Del Rey et al., 2006). Here, IL-1RA (10 mg/kg) showed only a very modest and temporary protective effect against 250 μg/kg LPS-induced decreases in glucose 2 h postchallenge and no effect thereafter, as detailed in Figure 1*G*. Therefore, systemic IL-1β is sufficient to lower blood glucose, but it is not indispensable for LPS-induced decreases in glucose.

### Blood glucose concentration is a major determinant of LPS-induced acute hypoactivity

**Figure 2.**
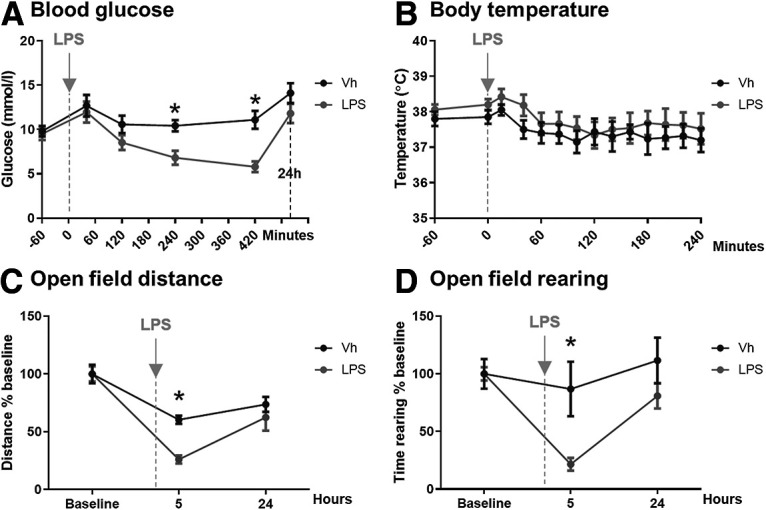
The impact of systemically applied LPS on blood glucose, body temperature and activity. ***A***, Blood glucose. Effect of systemic LPS on blood glucose levels from 60 min before the challenge to 24 h after it. LPS (250 µg/kg, i.p.; *n* = 8) induced a significant decrease of blood glucose levels at 240 min (*p* = 0.0206) and 420 min (*p* = 0.007) when compared with vehicle-treated (Vh) animals (*n* = 8). Main effect of treatment (*F*_(1,14)_ = 9.74; *p* = 0.0075) and time (*F*_(3,40)_ = 11.09). No difference was found at 24 h after LPS (*p* = 0.66). ***B***, Body temperature. Effect of systemic LPS challenge (250 µg/kg, i.p.; *n* = 5) on body temperature as measured using subcutaneous temperature transponders. No differences were found when compared with vehicle-treated (Vh) animals (*n* = 6). Main effect of time (*F*_(3,24)_ = 5.166; *p* = 0.0082). ***C***, Open field distance. Effects of systemic LPS in the open field test. LPS (250 µg/kg, i.p.; *n* = 8) significantly decreased the traveled distance at 5 h (*p* < 0.0001) when compared with vehicle-treated (Vh) group (*n* = 12). Main effect of treatment (*F*_(1,18)_ = 6.43; *p* = 0.0207) and time (*F*_(1,22)_ = 29.46; *p* < 0.0001). ***D***, Open field rearing. Time spent rearing was significantly decreased by LPS (250 µg/kg, i.p.; *n* = 8) at 5 h (*p* = 0.0266) in comparison with vehicle-treated (Vh) mice (*n* = 12). Main effect of treatment (*F*_(1,15)_ = 10.11; *p* = 0.0062) and time (*F*_(1,20)_ = 9.458; *p* = 0.0035). All annotated Bonferroni *post hoc* tests were performed after significant main effects or interactions in ANOVA analysis: *p < 0.05. All data are expressed as mean ± SEM.

IL-1β has been reported as the major driver of sickness behavior (Matsuwaki et al., 2017; Dantzer, 2018). We sought to understand whether IL-1β signaling or decreases in glucose might be the proximate cause of LPS-induced hypoactivity. We first showed that LPS, at 250 µg/kg, intraperitoneally, produces an acute decrease in blood glucose, emerging from ∼2 h and statistically significant at 4 h (*p* = 0.0206) and 7 h (*p* = 0.0074), that temporally overlaps with acute suppression of locomotor activity (*p* = 0.0207) and rearing behavior (*p* = 0.0062). This hypoglycemia and inactivity are not occurring in the context of elevated body temperature, which does not change significantly during this period (Fig. 2).

**Figure 3.**
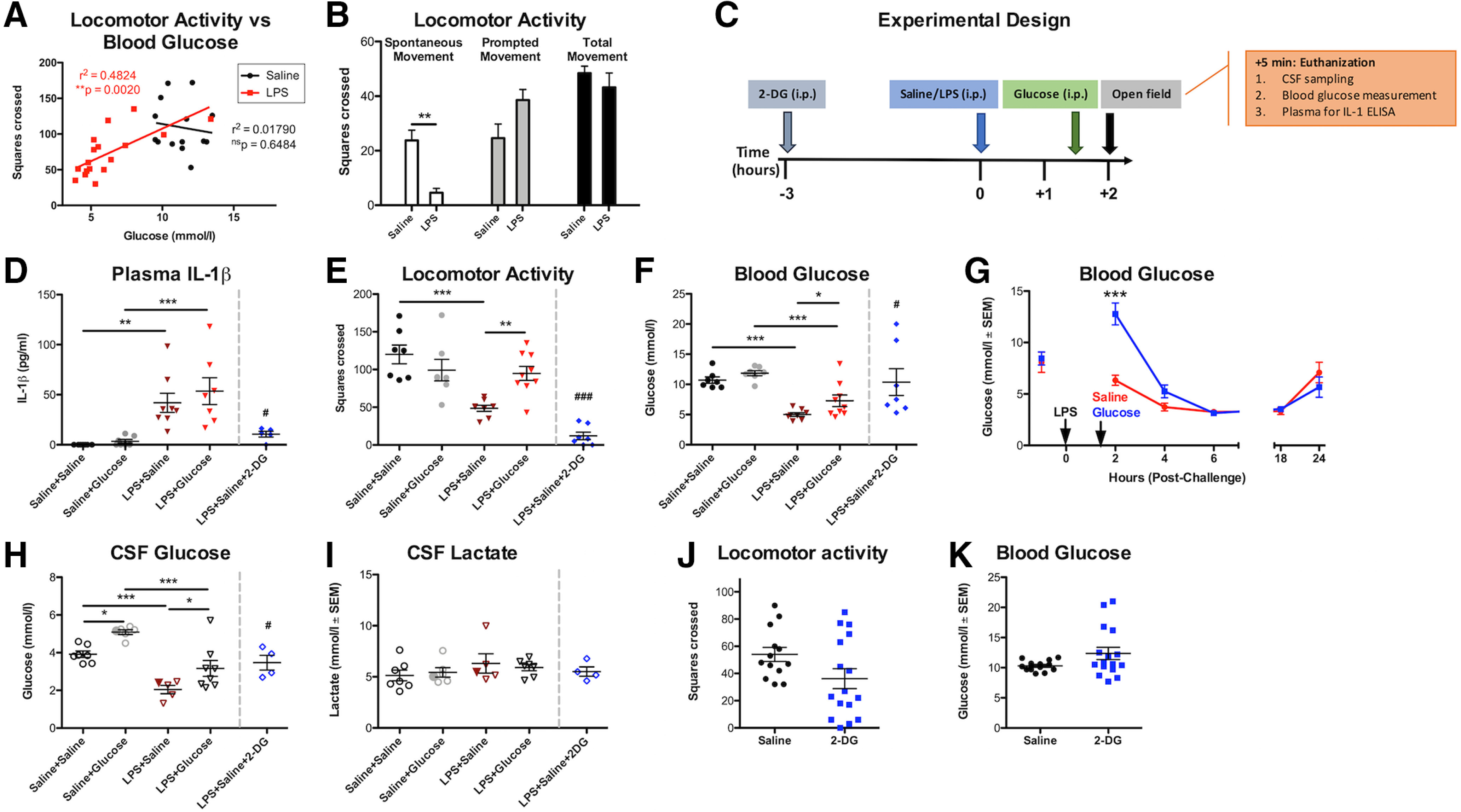
Low blood glucose concentration drives LPS-induced hypoactivity. ***A***, Linear regression analyses of locomotor activity (squares crossed/3 min) versus blood glucose concentration (mmol/l) in animals challenged with saline (*n* = 14) and LPS (*n* = 17). Blood glucose concentrations significantly correlated with locomotor activity in LPS-treated mice. ***B***, LPS significantly reduces spontaneous activity in the open field compared with saline-treated controls. Prompting inactive mice to move by gently nudging them with a fingertip results in similar levels of activity, showing that LPS mice are capable of moving but are not motivated to do so. ***C***, Timeline for treatments and sampling times. Glucose (2 g/kg, i.p.) was administered 1.5 h post-LPS challenge (250 µg/kg, i.p.), and open field behavior was measured 2 h post-LPS challenge. Five minutes after open field testing, mice were euthanized, CSF samples taken, blood glucose levels assessed, and plasma collected for IL-1β ELISA. In one group, 2-DG (2 g/kg, i.p.) was given 3 h before LPS. ***D***, LPS (250 μg/kg, i.p.; *n* = 8) induced IL-1β production (*F*_(1,25)_ = 29.88; *p* < 0.001), which was unaffected by glucose co-administration (*n* = 7; 90 min post-LPS) but blocked by 2-DG administration (intraperitoneal, *n* = 5, #*p* = 0.0296 vs LPS+saline). ***E***, Locomotor activity was suppressed by LPS (main effect of LPS: *F*_(1,27)_ = 13.39; *p* = 0.0011) but rescued by glucose co-administration (interaction between treatments: *F*_(1,27)_ = 10.48; *p* = 0.0032); **significant difference between LPS+glucose (*n* = 9) and LPS+saline (*n* = 8), and these were not significantly different to saline+saline (*n* = 7) or saline+glucose controls (*n* = 7). 2-DG+LPS completely suppressed locomotor activity in LPS-treated mice (*t*_(13)_ = 5.766; ###*p* < 0.0001 vs LPS+saline). ***F***, Blood glucose was suppressed by LPS (main effect: *F*_(1,27)_ = 60.00; *p* < 0.0001) and modestly increased by glucose (main effect: *F*_(1,27)_ = 6.721; *p* = 0.0152), and *post hoc* tests showed that LPS+glucose was significantly different to LPS+saline. ***G***, Glucose treatment 1.5 h after LPS provided significant but transient protection against LPS-induced hypoglycemia. ***H***, CSF (from the same animals) showed a main effect of LPS (*F*_(1,22)_ = 39.85; *p* < 0.0001) and a strong main effect of glucose (*F*_(1,22)_ = 14.57; *p* = 0.0009). LPS+glucose was significantly different to LPS+saline in *post hoc* analysis (*p* < 0.05). Two data points in these analyses represent two pooled samples each (in the saline+glucose and LPS+saline groups where some CSF samples were too low in volume to be assessed). They have been highlighted as slightly larger, filled symbols. ***I***, CSF lactate levels (same animals) were not altered by the treatments described. Again, the same samples were pooled for this analysis. In LPS-naive mice, 2-DG on its own does not significantly affect (***J***) spontaneous activity nor does it: have any effect on (***K***) blood glucose. Significance levels for Bonferroni *post hoc* tests: *p < 0.05, **p < 0.01, ***p < 0.001. All data are expressed as mean ± SEM.

LPS-induced hypoactivity in c57BL/6J mice (squares crossed/3 min) was significantly positively correlated with blood glucose levels (Fig. 3*A*), with low blood glucose predicting low activity in LPS-treated mice (*r*^2^ = 0.4824, *p* = 0.002), while no significant correlation was present between blood glucose and activity in saline-treated animals. In a separate experiment, LPS-treated mice showed significantly less spontaneous activity in the open field compared with saline-treated controls but when inactive mice were prompted to move, using a gentle finger nudge, similar levels of activity were observed in both groups (Fig. 3*B*) demonstrating that these animals are not unable to move or prevented from moving due to reduced energy availability. Rather, LPS-induced hypoactivity (Fig. 3*A*) reflects reduced spontaneous activity. Moreover, the changes in blood glucose and activity are not dependent on changes in body temperature since parallel experiments (Fig. 2*B*) demonstrated that body temperature was not significantly elevated under these conditions.

We then hypothesized that LPS-induced hypoactivity would be mitigated by treatment with glucose (2 g/kg, i.p.). In our experimental design (Fig. 3*C*), we also included a separate group of mice that received LPS+2-DG, which inhibits glucose-6-phosphate isomerase to prevent glycolysis, which in turn blocks macrophage synthesis of IL-1β (Tannahill et al., 2013). LPS robustly produced IL-1β, reduced blood and CSF glucose levels, and suppressed activity (Fig. 3*D–H*). Glucose treatment had no effect on IL-1β production (Fig. 3*D*) but significantly improved locomotor activity (Fig. 3*E*) and increased circulating glucose concentration (Fig. 3*F*). Administering glucose to mice, following LPS treatment, only transiently protected against LPS-induced decreases in blood glucose levels (Fig. 3*G*) as has previously been shown (Del Rey et al., 2006). In CSF, LPS significantly reduced glucose concentrations, and similar to the periphery, glucose treatment significantly protected against this (Fig. 3*H*). However, CSF lactate remained statistically unchanged in all groups (Fig. 3*I*). Therefore, LPS reduces CSF glucose concentration by ∼50%, and this can be mitigated by systemic glucose administration, with concomitant rescue of spontaneous activity. As predicted, 2-DG blocked LPS-induced IL-1β secretion (Fig. 3*D*), and yet hypoactivity remained striking (Fig. 3*E*). Therefore, animals with high IL-1β can remain spontaneously active if glucose concentration is temporarily boosted, and despite LPS producing no IL-1β when animals were additionally exposed to an inhibitor of glycolysis, these 2-DG-treated LPS animals show even less locomotor activity. These findings were replicated in a separate cohort of mice using a separate batch of LPS (100 μg/kg; i.p.; data not shown).

While 2-DG had a significant effect on spontaneous activity and blood glucose in LPS-treated mice, 2-DG on its own only modestly reduced locomotor activity (Fig. 3*J*), and this decrease was not statistically significant (*t* = 1.894; df = 27, *p* = 0.069) in this normally distributed dataset. Likewise, 2-DG had no significant impact on blood glucose concentrations (Mann–Whitney, *U* = 74.00; *p* = 0.1951). However, these data were not normally distributed (failed 2/3 normality tests), and a small number of samples showed higher levels (Fig. 3*K*). On subjective examination, mice receiving 2-DG alone did not appear sick but those showing the lowest activity post-2-DG tended to be those showing the highest levels of glucose, perhaps suggesting that blocking glucose utilization (functionally similar to hypoglycemia) could increase blood glucose but still produce inactivity as has been shown with higher doses of 2-DG (Voss et al., 2018).

Collectively, the data demonstrate that reduced glucose metabolism is a key proximate cause of LPS-induced suppression of spontaneous activity.

### Neurodegeneration increases susceptibility to cognitive impairments due to reduced glucose availability

**Figure 4.**
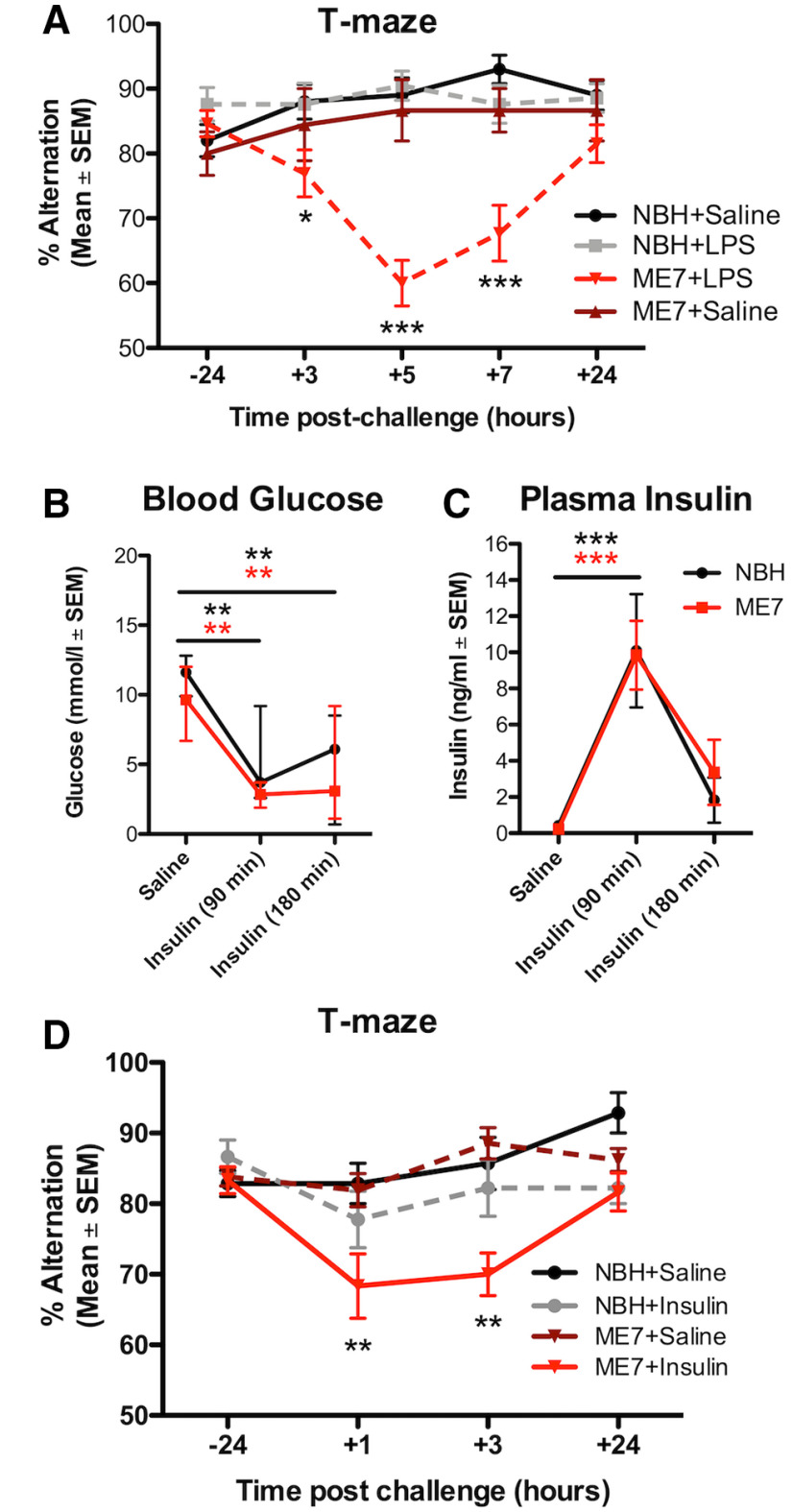
Insulin-induced reductions in blood glucose produces acute cognitive dysfunction selectively in mice with prior neurodegeneration. ***A***, ME7 mice have a cognitive vulnerability under LPS treatment (*n* = 26) that was not present in NBH mice treated with LPS (*n* = 21). Saline does not induce cognitive deficits either in NBH (*n* = 20) or in ME7 (*n* = 9) mice. There was an interaction between treatment group and time (*F*_(12,288)_ = 5.00; *p* < 0.0001). Blood glucose (mmol/l; ***B***) and plasma insulin concentrations (***C***) in saline-treated or insulin-treated (11.5 IU/kg, i.p.) NBH and ME7 mice. There were similar reductions in blood glucose (***B***, main effect of insulin, *F*_(2,20)_ = 17.11; *p* < 0.0001) and equivalent insulin concentrations over 180 min in ME7 and NBH animals (***C***, main effect of insulin, *F*_(2,28)_ = 22.86; *p* < 0.0001). ***D***, T-maze alternation in ME7 and NBH mice postchallenge with saline or insulin (+1 h = 40–160 min; and +3 h = 160–300 min postinsulin). Testing was performed earlier than in LPS-treated mice as insulin produces a more rapid decrease in blood glucose. There was a significant main effect of insulin (*F*_(3,135)_ = 7.418; *p* = 0.0004) and an interaction of ME7 and insulin (*F*_(9,135)_ = 3.050; *p* = 0.0024). ME7+insulin-treated mice (*n* = 12) had significantly lower alternation scores compared with NBH+saline controls (*n* = 7) at 1 and 3 h post-injection (NBH+insulin: *n* = 9; ME7+saline: *n* = 13). All data expressed as mean and standard error of the mean (SEM). Significance levels for Bonferroni post hoc tests: *p < 0.05, **p < 0.01, ***p < 0.001.

We have previously shown, using the ME7 model, that evolving neurodegeneration progressively increases susceptibility to LPS-induced transient working memory impairments on a T-maze task (Murray et al., 2012; Skelly et al., 2019). We replicate this here to illustrate the time course of these changes, and confirm that LPS does not produce such deficits in normal animals (Fig. 4*A*). We hypothesized that this cognitive vulnerability in ME7 mice may be explained by a greater tendency toward metabolic insufficiency and that cognitive function in ME7 mice might be less able to cope with limiting glucose. We tested this hypothesis using insulin (11.5 IU/kg, i.p.), which significantly lowered blood glucose in both ME7 and NBH mice (Fig. 4*B*). Basal levels of insulin were equivalent in ME7 and NBH mice, and all mice showed similar insulin pharmacokinetics on insulin treatment (Fig. 4*C*). Despite this, and analogous to LPS-induced cognitive deficits (Fig. 4*A*), insulin induced significant acute working memory dysfunction in ME7 mice that was absent in NBH controls (Fig. 4*D*).

**Figure 5.**
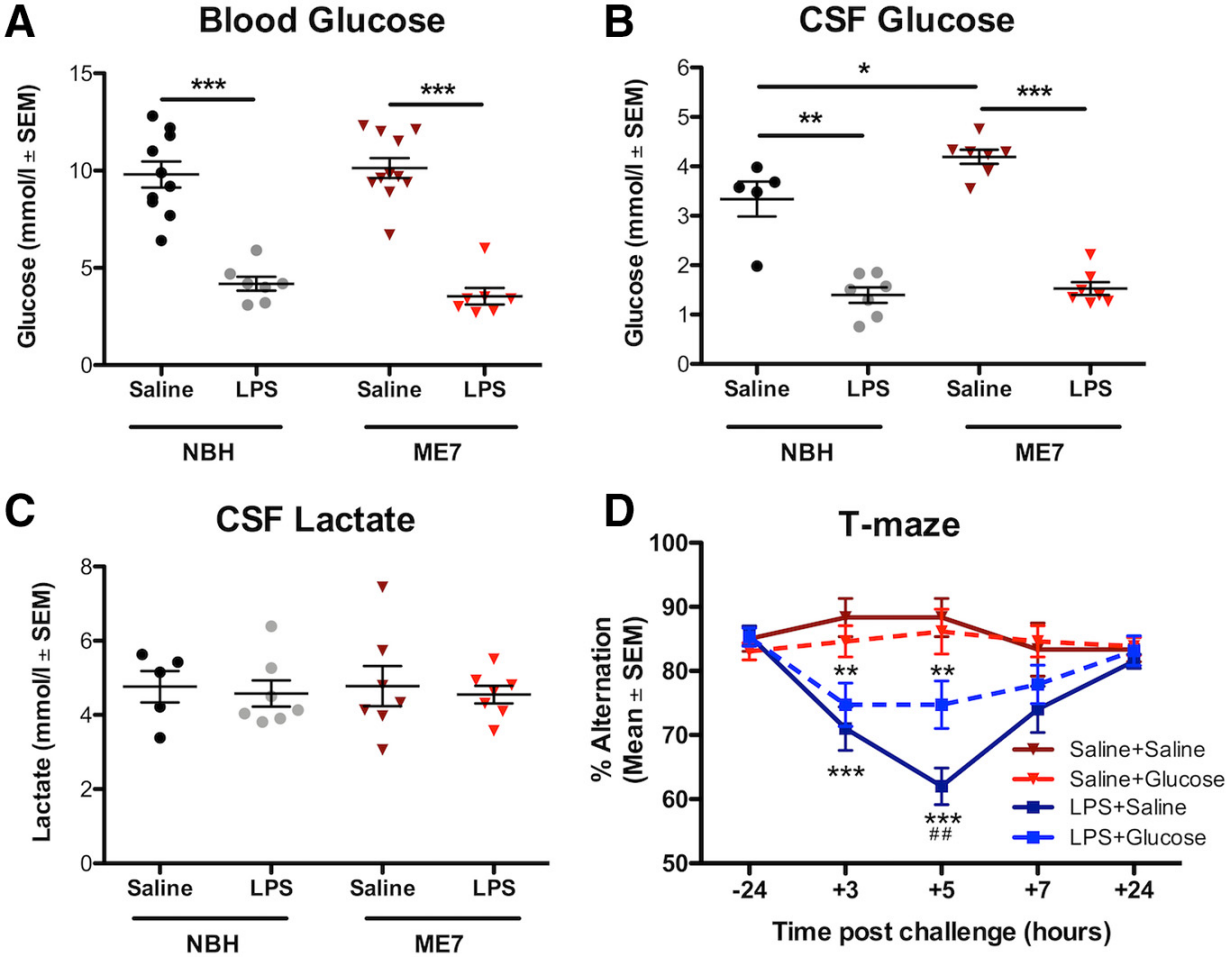
LPS-induced cognitive dysfunction in mice with prior neurodegeneration can be ameliorated by glucose administration. After 5 h, LPS produced equivalent decrease in glucose concentration in blood (***A***) and CSF glucose in ME7 (*n* = 7) and NBH mice (*n* = 7; ***B***) compared with their respective saline-treated controls (*n* = 11 and *n* = 10, respectively). There were main effects of LPS on blood glucose (*F*_(1,31)_ = 118.3; *p* < 0.0001) and on CSF glucose (*F*_(1,22)_ = 146.5; *p* < 0.0001) and also an effect of disease on CSF glucose (*F*_(1,22)_ = 6.665; *p* = 0.0170), with ME7+saline > NBH+saline by *post hoc* analysis. ***C***, There were no differences in CSF lactate levels. ***D***, T-maze alternation in ME7 mice postchallenge with saline or LPS, co-treated with glucose (2 g/kg) or saline. LPS+saline group (*n* = 20) showed robust cognitive impairment, but the LPS+glucose group (*n* = 19) showed significant attenuation. Two-way repeated measures ANOVA showed a main effect of LPS (*F*_(3,240)_ = 13.75; *p* < 0.0001) and an interaction of LPS and glucose (*F*_(12,240)_ = 3.740; *p* < 0.0001). LPS+glucose mice performed significantly better than the LPS+saline group at 5 h postchallenge (^##^*p* < 0.01). Significance levels for Bonferroni *post hoc* tests: *p < 0.05, **p < 0.01, ***p < 0.001.

Given the ability of insulin-induced hypoglycemia to trigger cognitive deficits selectively in mice with existing neurodegenerative disease (ME7), we examined whether LPS produced differential hypoglycemic responses in NBH and ME7 animals. Mice were inoculated with ME7 or NBH and, 16 weeks later, challenged with saline or LPS (100 µg/kg, i.p.). LPS produced similar glucose reductions in NBH and ME7 mice in both blood (Fig. 5*A*) and in CSF (Fig. 5*B*), although baseline CSF glucose concentration was slightly higher in ME7 animals with respect to NBH. CSF lactate levels were similar in all four groups (Fig. 5*C*). Since ME7 and NBH mice showed equivalent reduction in glucose, but differential cognitive outcomes post-LPS (Fig. 4*A*), and because LPS-induced sickness behavior can be reversed byintraperitoneal glucose (Fig. 3*E*), we hypothesized that the LPS-induced cognitive impairment in ME7 mice might be mediated by limiting glucose supply/utilization. ME7 mice were trained on the “escape from water” T-maze, until criterion performance of >80% correct was achieved. They were then treated with saline or LPS (100 μg/kg, i.p.) and, 2.5 h after LPS, treated with saline or glucose (2 g/kg, i.p.) before undergoing T-maze testing. Neither saline-treated nor glucose-treated ME7 mice deviated from baseline T-maze performance in the absence of LPS, but LPS-treated ME7 mice showed robust impairment between 3–7 h post-LPS. Those impairments in ME7+LPS+saline mice were significantly attenuated by glucose applied 2.5 h after LPS (significant interaction of treatment and time: *F*_(12,240)_ = 3.740; *p* < 0.0001; Fig. 5*D*). Bonferroni *post hoc* analysis showed that ME7+LPS+glucose mice were significantly less impaired than ME7+LPS+saline mice at 5 h (*p* < 0.01).

### Human delirium triggered by acute inflammatory trauma (hip fracture) is associated with altered carbohydrate metabolism

Acute inflammation disrupted glucose metabolism and this caused acute cognitive dysfunction (Fig. 5). We have previously demonstrated that this LPS-induced cognitive deficit is acute, transient, and fluctuating, occurs only in animals with prior degenerative pathology, and represents the best validated animal model of delirium superimposed on dementia (Davis et al., 2015; Schreuder et al., 2017). Therefore, seeking to investigate generalizability of these findings from mice, we assessed CSF concentrations of glycolytic metabolites in a cohort of acute hip fracture patients admitted for hip fracture repair with spinal anesthesia (for patient information, see Table 2). This represents an ideal cohort because CSF sampling is possible at the time of spinal anesthesia and because delirium occurs in a significant subset of these patients, and an acute inflammatory trauma (fracture) has been the proximate trigger for this delirium (Hall et al., 2018).

**Table 2.** Demographic information for patients recruited to the study

	No delirium (*n* = 32)	Prevalent delirium (*n* = 40)	*p* valued^d^		No dementia (*n* = 55)	Dementia (*n* = 59)	*p* value
Median age, years (range)	84.5 (60–93)	85 (68–95)	0.69		84 (60–101)	86 (64–96)	0.25
Male, *n* (%)	8 (25)	11 (27.5)	0.81		16 (29)	15 (25)	0.66
**Dementia, *n* (%)**^a^	2 (6.3)	32 (80)	<0.001	**Prevalent delirium, *n* (%)**	8 (15)	32 (54)	<0.001
Independent in activities of daily living, *n* (%)^b^	23 (71.9)	8 (20)	<0.001		39 (71)	7 (12)	<0.001
Living in an institution, *n* (%)	3 (9.4)	20 (50)	<0.001		3 (6)	37 (63)	<0.001
APACHE II, median (IQR)^c^	8 (6.3–9.8)	9 (8–11)	0.004		8 (7–10)	9 (8–10)	0.76
CCI, median (IQR)	1 (0–1.8)	1 (0–2)	0.044		1 (0–2)	1 (0–2)	0.58
ASA score, median (IQR)	2 (2–3)	3 (3–3)	<0.001		3 (2–3)	3 (2–3)	0.33

*^a^*Based on consensus in an expert panel.

*^b^*Defined as 19 or 20 points on Barthel activities of daily living.

*^c^*Without information on hematocrit and arterial blood gas.

*^d^*Mann–Whitney test and χ^2^ tests depending on data distribution.

APACHE II = acute physiology and chronic health evaluation II; ASA = American Society of Anesthesiologists Physical Health Classification; CCI = Charlson Comorbidity Index score; IQR = interquartile range. The columns on the left (no delirium vs prevalent delirium) contain all patients with prevalent delirium and those who never experienced delirium during their hospital stay. The columns on the right (no dementia vs dementia) contain a larger number of patients since they include also patients who were subsyndromal for delirium and those who may have experienced delirium at some later point in their hospitalization (but did not have delirium prevalent at the time of CSF sampling).

**Figure 6.**
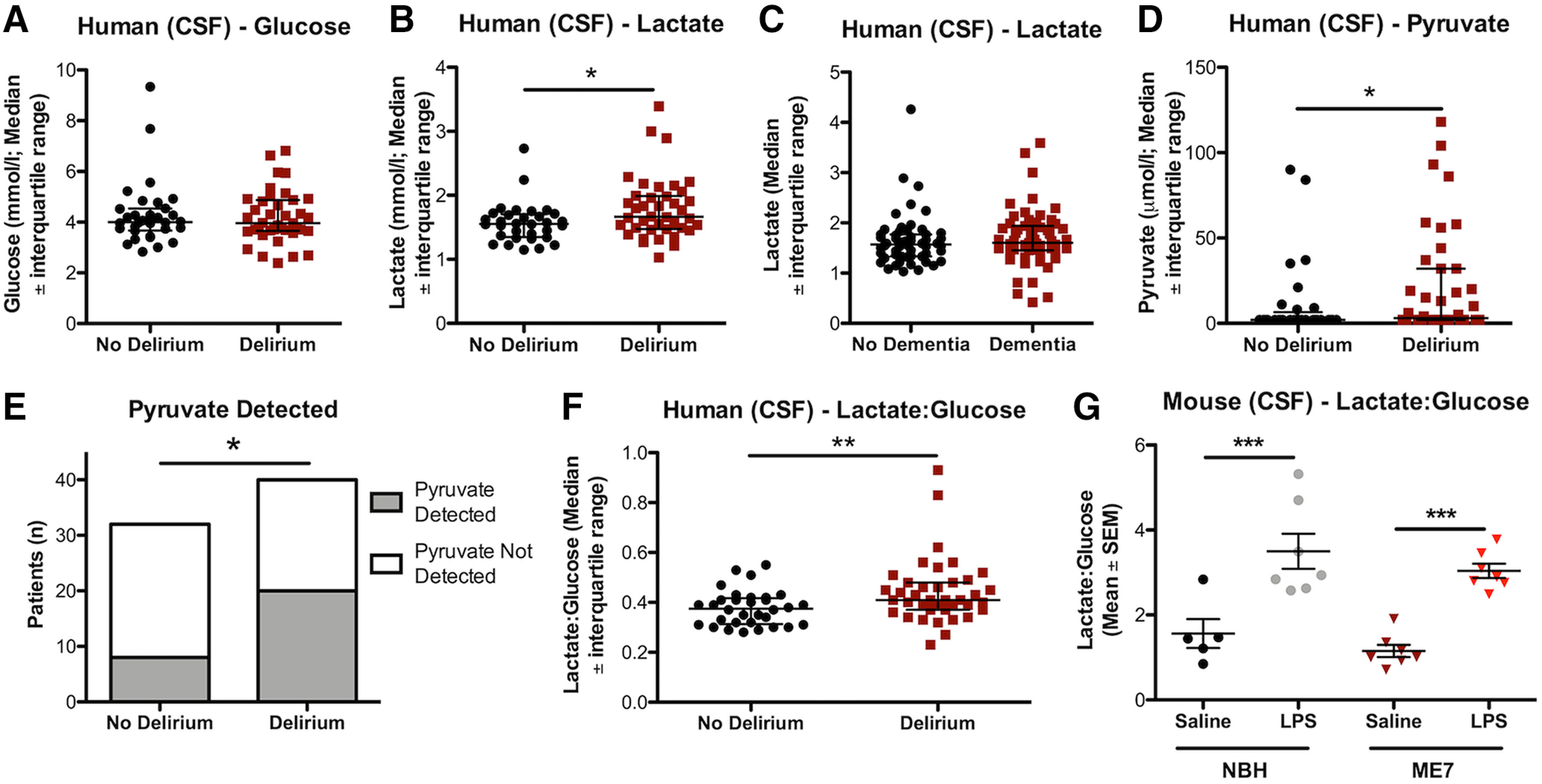
Derangement of energy metabolism in human delirium. Metabolite levels in the CSF of hip fracture patients with delirium (*n* = 40) at the time of CSF sampling compared with age-matched patients with no delirium at any point of their hospital stay (*n* = 32). ***A***, Glucose levels in delirium (*n* = 39, one sample omitted due to a read error) and non-delirium cases were not significantly different (Mann–Whitney *U* = 606.5; *p* = 0.8442). ***B***, Patients with delirium had significantly higher levels of lactate in their CSF compared with controls (*U* = 442.5; *p* = 0.0128). ***C***, Lactate levels in the CSF of hip fracture patients with dementia (*n* = 59) at the time of CSF sampling compared with age-matched patients with no dementia (*n* = 55; no significant difference in CSF lactate *U* = 1438; *p* = 0.2954). ***D***, Patients with delirium showed significantly higher pyruvate levels compared with controls (*U* = 514.5; *p* = 0.0494), with all levels below the minimum detectable level (4 µmol/l) entered as 50% of this LOD (i.e., 2 µmol/ml). ***E***, In addition, pyruvate was detected significantly more often in patients with delirium compared with patients without delirium at time of CSF sampling (Fisher's exact test, *p* = 0.0306). ***F***, The LGR for patients with delirium (*n* = 39) was significantly higher compared with controls (*U* = 399.5; *p* = 0.0048). ***G***, LPS significantly increased the CSF LGR in both ME7 (*n* = 7) and NBH mice (*n* = 7) compared with their respective saline-treated controls (*n* = 7 and *n* = 5; *F*_(1,22)_ = 44.58; *p* < 0.0001). Significance levels for Mann-Whitney U tests (***B***, ***D*** and ***F***) are annotated by *p < 0.05, **p < 0.01, for Fisher's exact test (***E***) by *p < 0.05 and for Bonferroni post-hoc tests (***G***) by ***p < 0.001.

Hip fracture patients with ongoing delirium at the time of lumbar puncture (“prevalent delirium”) were compared with those without any signs of delirium during hospital stay (preoperatively and postoperatively) on CSF glucose, lactate, and pyruvate (commonly used markers of central energy metabolism disturbance in clinical populations; Leen et al., 2012; Zhang and Natowicz, 2013). CSF glucose was not different in those with and without delirium (Fig. 6*A*). A previous study of all-cause delirium versus stable dementia (Caplan et al., 2010) provided the a priori hypothesis that delirium would be associated with elevated lactate, and lactate was indeed significantly elevated during delirium (one-tailed Mann–Whitney analysis; *p* = 0.0128; Fig. 6*B*). Changes in CSF lactate, associated with delirium, were not explained by dementia status. That is, comparing all patients with a diagnosis of dementia to all patients without dementia, in an expanded cohort (see Table 2), revealed that lactate levels in the CSF of hip fracture patients with dementia (*n* = 59), compared with age-matched patients with no dementia (*n* = 55), were not significantly different (*U* = 1438, *p* = 0.2954; Fig. 6*C*). Median pyruvate levels were significantly elevated in delirium (Fig. 6*D*). Although pyruvate was not detectable in CSF of all patients, it was detected significantly more often in patients with delirium (Fig. 6*E*). The difference between delirium and no-delirium with respect to frequency of pyruvate detection was found to be significant (*p* = 0.0306) using the Fisher's exact test.

Increases in the CSF lactate:glucose ratio (LGR) have been associated with reduced consciousness (Sanchez et al., 2013) and increased mortality (Lozano et al., 2020), and here, these data indicate an elevated LGR both in humans experiencing delirium after acute inflammatory trauma (Fig. 6*F*) and in mice cognitively impaired by acute systemic inflammation (Fig. 6*G*). The changes in LGR observed in mice and humans differ in how they arise, with an increase in the ratio driven by increases in lactate in humans (Fig. 6*A*,*B*) and by decreases in glucose in the mouse (Fig. 5*A*,*C*), but both mouse and human datasets indicate that there is a significant derangement of brain energy metabolism following these inflammatory insults, and in mice, this is clearly causal for acute cognitive dysfunction.

## Discussion

We demonstrate that LPS-induced hypoglycemia suppresses spontaneous activity in mice. Glycemic status was a major determinant of spontaneous activity after LPS. Reduced glucose availability also drove LPS-induced acute cognitive impairment in mice with underlying neurodegeneration and impairments were mitigated by exogenous glucose. The degenerating brain is also selectively vulnerable to cognitive disruption by insulin, despite equivalent blood glucose reductions. Finally, inflammatory trauma-induced delirium in humans was associated with altered central energy metabolism.

### Hypoactivity

Reducing blood glucose can be adaptive for the organism, depriving infectious agents of a key fuel source, and providing further glucose can actually increase *Listeria monocytogenes*-induced mortality (Wang et al., 2016). Nonetheless, we show, in the acute phase, that significant LPS-induced decreases in blood glucose reduce CSF glucose and suppress spontaneous activity. This is consistent with prior studies showing correlation between blood glucose and sickness behavior (Carlton and Demas, 2017) and others showing that insulin-induced hypoglycemia suppresses social activity in c57BL/6 mice (Park et al., 2008, 2012). Here, by directly increasing glucose availability, by applying exogenous glucose, we prevented LPS-induced suppression of activity without reducing IL-1β. Moreover, 2-DG completely blocked LPS-induced IL-1β secretion, as previously shown for macrophage IL-1 production (Tannahill et al., 2013) but in also preventing glucose utilization it further suppressed activity. Although IL-1 is widely implicated in LPS-induced sickness behavior, LPS-induced hypoactivity persisted even when IL-1 secretion or action was blocked (IL-1RA, IL-1RI^−/−^). Although IL-1RA protection inhibited LPS-induced hypoglycemia in prior studies, those effects were partial: 100 µg IL-1RA/mouse marginally mitigated hypoglycemia (Del Rey et al., 2006), just as we observed here. IL-1RA (at 200 µg/mouse) completely blocked effects of 25 µg/kg IL-1β (Fig. 1*D*), which leads to blood levels of ∼600 pg/ml IL-1β (Skelly et al., 2013), and this IL-1RA dose should therefore block the effects of LPS-induced IL-1β (∼50–100 pg/ml; Teeling et al., 2007; Murray et al., 2011; Skelly et al., 2013). However, TNF-α also triggers hypoglycemia (Oguri et al., 2002); thus, IL-1RA can have only a partial effect in limiting LPS-induced hypoglycemia. Therefore, while IL-1β might be a key mediator of hypoglycemia at 25 µg/kg LPS (Del Rey et al., 2006), IL-1RA barely limits hypoglycemia with LPS at 250 µg/kg (current study). Ultimately, the ability of glucose to restore activity in the current experiments reveals the importance of glucose uptake and use in fueling and regulating spontaneous activity under LPS-induced inflammation.

The hypothalamus monitors levels of circulating IL-1β (Matsuwaki et al., 2017) and glucose (López-Gambero et al., 2019) and coordinates sickness behavior. IL-1β action in the hypothalamus is proposed to reprogram the organism to operate at lower circulating glucose levels after LPS (25 µg/kg; Del Rey et al., 2006), and these authors propose that IL-1β increases brain energy metabolism (Del Rey et al., 2016). [^18^F]-fluorodeoxyglucose (FDG-PET) experiments show that high-dose LPS (15 mg/kg) increased hypothalamic activity (Wang et al., 2016) but LPS (10 mg/kg, i.p.) has been shown to decrease glucose uptake across multiple cortical regions (Semmler et al., 2008). If, as reported, IL-1 lowers the set-point for glucose homeostasis, allowing animals to function efficiently at lower glucose concentrations (Del Rey et al., 2006, 2016; Besedovsky and Del Rey, 2010), it is not intuitive why transiently increasing available glucose should rapidly increase spontaneous activity. Here, we show that administration of glucose raises both blood and CSF glucose (Fig. 3), and although this “top-up” of glucose provides only temporary and partial increases in available glucose (Fig. 3*G*; Del Rey et al., 2006), this is sufficient to restore spontaneous activity and cognition. We therefore propose that while the hypothalamus might be selectively active during acute inflammation, to coordinate neuroendocrine responses to the acute threat, the suppression of spontaneous locomotor activity that is actually observed may reflect decreased neural activity underpinned by decreased available glucose.

The neuroanatomical basis of LPS-induced suppression of exploratory activity is incompletely understood but correlates with suppression in cFOS in brain areas associated with positive motivation (Stone et al., 2006) and exploratory behavior (Gaykema and Goehler, 2011). LPS triggers norepinephrine (NE) release in the hypothalamus (Francis et al., 2001) and lesioning caudal medullary NE inputs to the hypothalamus blocks LPS-induced hypoactivity (Gaykema and Goehler, 2011). Hypoglycemia, hyperinsulinemia (Beverly et al., 2001), and 2-DG treatment (Beverly et al., 2000) all induce hypothalamic NE release, suggesting potential points of convergence for how inflammation and impaired glucose metabolism may drive changes in behavior during sickness.

Whatever the neuroanatomical and neurotransmitter underpinnings, the current data strongly support the idea that available and usable glucose is a key determinant of LPS-induced suppression of activity. This has implications for studies using peripheral LPS to examine theneurophysiological and behavioral consequences of systemic infection. Levels of circulating LPS arising from bolus LPS challenges are higher than in active infection (Danner et al., 1991). Bolus LPS treatment (4 ng/kg, i.v.) in human volunteers transiently decreases plasma glucose (Bloesch et al., 1993) while active infection typically does not produce hypoglycemia (Furman et al., 1988). Therefore, although bolus LPS would appear to have face validity as a model of systemic infection, if key behavioral and neurophysiological changes induced by LPS in experimental subjects are underpinned by a physiological change, i.e., hypoglycemia, that rarely occurs during active infection, this necessitates a review of the generalizability of bolus LPS-induced changes to understand changes during active infection.

### Acute cognitive dysfunction and delirium

Human data suggest that reduced glucose uptake in the medial temporal lobe associates with impaired performance in hippocampal-dependent tasks (Harrison et al., 2014). Remarkably, despite the robust and long lasting reductions in available glucose shown here, normal LPS-treated mice maintain good working memory (Fig. 4; Skelly et al., 2019). However, the same decreases in glucose, caused by LPS or insulin, were sufficient to triggerdysfunction in animals with prior neurodegeneration. Exogenously added glucose does not improve cognition in young rats (Kealy et al., 2017) but enhances cognition in aged rats (McNay and Gold, 2001), supporting the idea that the same task may require additional metabolic support in aging/degenerating brain. We propose that the circuitry underpinning working memory may, during neurodegeneration, be operating close to thresholds for decompensation and may need to recruit additional brain areas to maintain this function. The addition of a further stressor may then be sufficient to unmask underlying vulnerability. Volunteers exposed to *Salmonella typhi* vaccination performed equally to controls on the Stroop test of executive function but recruited additional areas of the prefrontal and anterior cingulate cortex to maintain performance during inflammation (Harrison et al., 2009). If increased connectivity is required to maintain performance during inflammation, then inflammatory insults may unmask vulnerability, when evolving neurodegeneration impairs connectivity (Davis et al., 2015). The ME7 model of delirium during dementia has, until now, been an exemplar for an inflammatory hypersensitivity (Murray et al., 2012), but these data show that these mice are also more vulnerable to “bioenergetic stressors.” Despite equivalent reductions in blood and CSF glucose, NBH animals are resilient to hypoglycemia-induced cognitive impairment but ME7 animals are vulnerable, whether induced by LPS or by insulin.

The brain is a metabolically demanding organ and it may be adaptive, for survival, to minimize energy use in the brain and preserve autonomic function at the expense of higher cortical function. Engel and Romano proposed that delirium is driven by a failure to meet the brain's energy requirements, regardless of the underlying cause (Engel and Romano, 2004). Hypoglycemia is sufficient, alone, to produce delirium and EEG slowing, and this is reversed by glucose administration (Engel and Romano, 1944). Small CSF studies support the idea of metabolic disturbances during delirium: patients with delirium have elevated CSF lactate compared with non-delirious Alzheimer's disease controls (Caplan et al., 2010) and [^18^F]FDG-PET studies show decreased glucose uptake (Haggstrom et al., 2017). The posterior cingulate cortex, which is associated with attention and arousal, was particularly affected and disrupting energy metabolism here could be important in delirium.

Although hypoglycemia in mice is not precisely defined, the ME7 blood glucose concentrations here remained just above the clinical threshold for moderate hypoglycemia (3.9 mmol/l; Cryer, 2017). Iatrogenic hypoglycemia is common in patients using insulin for diabetes (Cryer, 2002) and is a major cause of emergency department admissions and adverse CNS effects in older patients (Shehab et al., 2016). Significantly, our ME7 data suggest that even when blood glucose levels do not fall into classical hypoglycemic ranges, these changes have significant deleterious impacts on brain function in those with prior vulnerability.

Importantly glucose was not lower in the hip fracture patients studied here (Fig. 6*A*). Both hypoglycemia and hyperglycemia increase risk for sepsis-associated encephalopathy (Sonneville et al., 2017), but hypoglycemia is less common. However, insulin insensitivity, and impaired glucose uptake, typically accompanies hyperglycemia-associated delirium. In humans, insulin resistance occurs on LPS (Agwunobi et al., 2000), infection (Virkamäki et al., 1992), and surgery (Thorell et al., 1994). Microcirculatory failure and tissue hypoxia, common in sepsis (Ince and Mik, 2016), may also disrupt glucose oxidation and the elevated lactate and pyruvate in the current study might indicate a shift from normal aerobic to anaerobic glycolysis. Although dementia status is a major risk factor for delirium (Davis et al., 2015), the changes in lactate and LGR observed are not explained by existing dementia. The data are consistent with previously demonstrated associations between delirium and increased CSF lactate (Caplan et al., 2010) and hypoxia (Tahir et al., 2018).

## Conclusion

Reduced glucose availability is a major driver of LPS-induced suppression of spontaneous activity. In animals made vulnerable by evolving neurodegeneration, this decreased glucose is now sufficient to trigger acute cognitive dysfunction indicating that metabolic insufficiency underlies cognitive dysfunction in this animal model resembling delirium. A disruption of energy metabolism also occurs in delirium triggered by inflammatory trauma. Together, the findings indicate that acutely disrupted energy metabolism likely contributes to general behavioral changes associated with sickness but also to acute neuropsychiatric disorders such as delirium. Systemic inflammation produces disproportionate brain dysfunction when superimposed on the vulnerable brain, as is evident during bacterial sepsis and infections such as SARS-CoV-2 in older people. The appropriate supply of both oxygen and energy substrates to the brain becomes especially important in those with existing cognitive vulnerability. The current data should focus attention on bioenergetic mechanisms of acute brain failure during acute illness and hospitalization in older adults.

## References

[B1] Agwunobi AO, Reid C, Maycock P, Little RA, Carlson GL (2000) Insulin resistance and substrate utilization in human endotoxemia. J Clin Endocrinol Metab 85:3770–3778. 10.1210/jcem.85.10.6914 11061537

[B2] American Psychiatric Association (2013) Neurocognitive disorders - delirium. In: Diagnostic and statistical manual of mental disorders, Ed 5, pp 596–601. Washington, DC: American Psychiatric Association.

[B3] Banks WA, Robinson SM (2010) Minimal penetration of lipopolysaccharide across the murine blood-brain barrier. Brain Behav Immun 24:102–109. 10.1016/j.bbi.2009.09.001 19735725 PMC2789209

[B4] Besedovsky HO, Del Rey A (2010) Interleukin-1 resets glucose homeostasis at central and peripheral levels: relevance for immunoregulation. Neuroimmunomodulation 17:139–141. 10.1159/000258707 20134186

[B5] Beverly JL, de Vries MG, Beverly MF, Arseneau LM (2000) Norepinephrine mediates glucoprivic-induced increase in GABA in the ventromedial hypothalamus of rats. Am J Physiol Regul Integr Comp Physiol 279:R990–R996. 10.1152/ajpregu.2000.279.3.R990 10956258

[B6] Beverly JL, De Vries MG, Bouman SD, Arseneau LM (2001) Noradrenergic and GABAergic systems in the medial hypothalamus are activated during hypoglycemia. Am J Physiol Regul Integr Comp Physiol 280:R563–R569. 10.1152/ajpregu.2001.280.2.R563 11208588

[B7] Bloesch D, Keller U, Spinas GA, Küry D, Girard J, Stauffacher W (1993) Effects of endotoxin on leucine and glucose kinetics in man: contribution of prostaglandin EII assessed by a cyclooxygenase inhibitor. J Clin Endocrinol Metab 77:1156–1163. 10.1210/jcem.77.5.8077306 8077306

[B8] Caplan GA, Kvelde T, Lai C, Yap SL, Lin C, Hill MA (2010) Cerebrospinal fluid in long-lasting delirium compared with Alzheimer's dementia. J Gerontol A Biol Sci Med Sci 65A:1130–1136. 10.1093/gerona/glq090 20530241

[B9] Carlton ED, Demas GE (2017) Glucose and insulin modulate sickness responses in male Siberian hamsters. Gen Comp Endocrinol 242:83–91. 10.1016/j.ygcen.2015.11.001 26542473 PMC4853293

[B10] Chakera AJ, Hurst PS, Spyer G, Ogunnowo-Bada EO, Marsh WJ, Riches CH, Yueh CY, Markkula SP, Dalley JW, Cox RD, Macdonald IA, Amiel SA, MacLeod KM, Heisler LK, Hattersley AT, Evans ML (2018) Molecular reductions in glucokinase activity increase counter-regulatory responses to hypoglycemia in mice and humans with diabetes. Mol Metab 17:17–27. 10.1016/j.molmet.2018.08.001 30146176 PMC6197723

[B11] Champy MF, Selloum M, Piard L, Zeitler V, Caradec C, Chambon P, Auwerx J (2004) Mouse functional genomics requires standardization of mouse handling and housing conditions. Mamm Genome 15:768–783. 10.1007/s00335-004-2393-1 15520880

[B12] Cryer PE (2002) Hypoglycaemia: the limiting factor in the glycaemic management of Type I and Type II diabetes. Diabetologia 45:937–948. 10.1007/s00125-002-0822-9 12136392

[B13] Cryer PE (2017) Individualized glycemic goals and an expanded classification of severe hypoglycemia in diabetes. Diabetes Care 40:1641–1643. 10.2337/dc16-1741 29162584

[B14] Cunningham C, Sanderson DJ (2008) Malaise in the water maze: untangling the effects of LPS and IL-1beta on learning and memory. Brain Behav Immun 22:1117–1127. 10.1016/j.bbi.2008.05.007 18640811 PMC4157220

[B15] Cunningham C, Maclullich AM (2013) At the extreme end of the psychoneuroimmunological spectrum: delirium as a maladaptive sickness behaviour response. Brain Behav Immun 28:1–13. 10.1016/j.bbi.2012.07.012 22884900 PMC4157329

[B16] Danner RL, Elin RJ, Hosseini JM, Wesley RA, Reilly JM, Parillo JE (1991) Endotoxemia in human septic shock. Chest 99:169–175. 10.1378/chest.99.1.169 1984950

[B17] Dantzer R (2004) Cytokine-induced sickness behaviour: a neuroimmune response to activation of innate immunity. Eur J Pharmacol 500:399–411. 10.1016/j.ejphar.2004.07.040 15464048

[B18] Dantzer R (2018) Neuroimmune Interactions: from the brain to the immune system and vice versa. Physiol Rev 98:477–504. 10.1152/physrev.00039.2016 29351513 PMC5866360

[B19] Davis DHJ, Skelly DT, Murray C, Hennessy E, Bowen J, Norton S, Brayne C, Rahkonen T, Sulkava R, Sanderson DJ, Rawlins JN, Bannerman DM, MacLullich AMJ, Cunningham C (2015) Worsening cognitive impairment and neurodegenerative pathology progressively increase risk for delirium. Am J Geriatr Psychiatry 23:403–415. 10.1016/j.jagp.2014.08.005 25239680 PMC4278840

[B20] Del Rey A, Roggero E, Randolf A, Mahuad C, McCann S, Rettori V, Besedovsky HO (2006) IL-1 resets glucose homeostasis at central levels. Proc Natl Acad Sci USA 103:16039–16044. 10.1073/pnas.0607076103 17035503 PMC1635123

[B21] Del Rey A, Verdenhalven M, Lörwald AC, Meyer C, Hernangómez M, Randolf A, Roggero E, König AM, Heverhagen JT, Guaza C, Besedovsky HO (2016) Brain-borne IL-1 adjusts glucoregulation and provides fuel support to astrocytes and neurons in an autocrine/paracrine manner. Mol Psychiatry 21:1309–1320. 10.1038/mp.2015.174 26643538

[B22] Draper A, Koch RM, van der Meer JW, Aj Apps M, Pickkers P, Husain M, van der Schaaf ME (2018) Effort but not reward sensitivity is altered by acute sickness induced by experimental endotoxemia in humans. Neuropsychopharmacology 43:1107–1118. 10.1038/npp.2017.231 28948979 PMC5854801

[B23] Elie M, Cole MG, Primeau FJ, Bellavance F (1998) Delirium risk factors in elderly hospitalized patients. J Gen Intern Med 13:204–212. 10.1046/j.1525-1497.1998.00047.x 9541379 PMC1496920

[B24] Engel GL, Romano J (1944) Delirium II. Reversibility of the electroencephalogram with experimental procedures. Arch Neurol Psychiatry 51:378–392. 10.1001/archneurpsyc.1944.02290280076004

[B25] Engel GL, Romano J (2004) Delirium, a syndrome of cerebral insufficiency. 1959. J Neuropsychiatry Clin Neurosci 16:526–538. 10.1176/jnp.16.4.526 15616182

[B26] Field RH, Gossen A, Cunningham C (2012) Prior pathology in the basal forebrain cholinergic system predisposes to inflammation-induced working memory deficits: reconciling inflammatory and cholinergic hypotheses of delirium. J Neurosci 32:6288–6294. 10.1523/JNEUROSCI.4673-11.2012 22553034 PMC3359617

[B27] Francis J, MohanKumar PS, MohanKumar SM (2001) Lipopolysaccharide stimulates norepinephrine efflux from the rat hypothalamus in vitro: blockade by soluble IL-1 receptor. Neurosci Lett 308:71–74. 10.1016/s0304-3940(01)01903-6 11457562

[B28] Furman BL, Walker E, Sidey FM, Wardlaw AC (1988) Slight hyperinsulinaemia but no hypoglycaemia in pertussis patients. J Med Microbiol 25:183–186. 10.1099/00222615-25-3-183 2894469

[B29] Gaykema RP, Goehler LE (2011) Ascending caudal medullary catecholamine pathways drive sickness-induced deficits in exploratory behavior: brain substrates for fatigue? Brain Behav Immun 25:443–460. 10.1016/j.bbi.2010.11.005 21075199 PMC3039108

[B30] Gibson QH, Swoboda BE, Massey V (1964) Kinetics and mechanism of action of glucose oxidase. J Biol Chem 239:3927–3934. 14257628

[B31] Griffin EW, Skelly DT, Murray CL, Cunningham C (2013) Cyclooxygenase-1-dependent prostaglandins mediate susceptibility to systemic inflammation-induced acute cognitive dysfunction. J Neurosci 33:15248–15258. 10.1523/JNEUROSCI.6361-11.2013 24048854 PMC3776067

[B32] Haggstrom LR, Nelson JA, Wegner EA, Caplan GA (2017) 2-(18)F-fluoro-2-deoxyglucose positron emission tomography in delirium. J Cereb Blood Flow Metab 37:3556–3567. 10.1177/0271678X17701764 28350285 PMC5669345

[B33] Hall RJ, Watne LO, Cunningham E, Zetterberg H, Shenkin SD, Wyller TB, MacLullich AMJ (2018) CSF biomarkers in delirium: a systematic review. Int J Geriatr Psychiatry 33:1479–1500. 10.1002/gps.4720 28585290

[B34] Harrison NA, Brydon L, Walker C, Gray MA, Steptoe A, Dolan RJ, Critchley HD (2009) Neural origins of human sickness in interoceptive responses to inflammation. Biol Psychiatry 66:415–422. 10.1016/j.biopsych.2009.03.007 19409533 PMC2885492

[B35] Harrison NA, Doeller CF, Voon V, Burgess N, Critchley HD (2014) Peripheral inflammation acutely impairs human spatial memory via actions on medial temporal lobe glucose metabolism. Biol Psychiatry 76:585–593. 10.1016/j.biopsych.2014.01.005 24534013 PMC4166523

[B36] Hennessy E, Gormley S, Lopez-Rodriguez AB, Murray C, Murray C, Cunningham C (2017) Systemic TNF-α produces acute cognitive dysfunction and exaggerated sickness behavior when superimposed upon progressive neurodegeneration. Brain Behav Immun 59:233–244. 10.1016/j.bbi.2016.09.011 27633985 PMC5176008

[B37] Horn T, Klein J (2010) Lactate levels in the brain are elevated upon exposure to volatile anesthetics: a microdialysis study. Neurochem Int 57:940–947. 10.1016/j.neuint.2010.09.014 20933036

[B38] Ince C, Mik EG (2016) Microcirculatory and mitochondrial hypoxia in sepsis, shock, and resuscitation. J Appl Physiol 120:226–235. 10.1152/japplphysiol.00298.2015 26066826

[B39] Inouye SK, van Dyck CH, Alessi CA, Balkin S, Siegal AP, Horwitz RI (1990) Clarifying confusion: the confusion assessment method. A new method for detection of delirium. Ann Intern Med 113:941–948. 10.7326/0003-4819-113-12-941 2240918

[B40] Irahara T, Sato N, Otake K, Matsumura S, Inoue K, Ishihara K, Fushiki T, Yokota H (2018) Alterations in energy substrate metabolism in mice with different degrees of sepsis. J Surg Res 227:44–51. 10.1016/j.jss.2018.01.021 29804861

[B41] Kealy J, Bennett R, Lowry JP (2015) Real-time effects of insulin-induced hypoglycaemia on hippocampal glucose and oxygen. Brain Res 1598:76–87. 10.1016/j.brainres.2014.12.015 25511995

[B42] Kealy J, Bennett R, Woods B, Lowry JP (2017) Real-time changes in hippocampal energy demands during a spatial working memory task. Behav Brain Res 326:59–68. 10.1016/j.bbr.2017.02.034 28249730

[B43] Leen WG, Willemsen MA, Wevers RA, Verbeek MM (2012) Cerebrospinal fluid glucose and lactate: age-specific reference values and implications for clinical practice. PLoS One 7:e42745. 10.1371/journal.pone.0042745 22880096 PMC3412827

[B44] Liu X, Nemeth DP, McKim DB, Zhu L, DiSabato DJ, Berdysz O, Gorantla G, Oliver B, Witcher KG, Wang Y, Negray CE, Vegesna RS, Sheridan JF, Godbout JP, Robson MJ, Blakely RD, Popovich PG, Bilbo SD, Quan N (2019) Cell-type-specific interleukin 1 receptor 1 signaling in the brain regulates distinct neuroimmune activities. Immunity 50:764–766. 10.1016/j.immuni.2019.02.012 30893590

[B45] López-Gambero AJ, Martínez F, Salazar K, Cifuentes M, Nualart F (2019) Brain glucose-sensing mechanism and energy homeostasis. Mol Neurobiol 56:769–796. 10.1007/s12035-018-1099-4 29796992

[B46] Lopez-Rodriguez AB, Hennessy E, Murray C, Lewis A, de Barra N, Fagan S, Rooney M, Nazmi A, Cunningham C (2018) Microglial and Astrocyte priming in the APP/PS1 model of Alzheimer's disease: increased vulnerability to acute inflammation and cognitive deficits. bioRxiv. doi: 10.1101/344218.

[B47] Lozano A, Franchi F, Seastres RJ, Oddo M, Lheureux O, Badenes R, Scolletta S, Vincent JL, Creteur J, Taccone FS (2020) Glucose and lactate concentrations in cerebrospinal fluid after traumatic brain injury. J Neurosurg Anesthesiol 32:162–169. 30893283 10.1097/ANA.0000000000000582

[B48] Mamad O, Islam MN, Cunningham C, Tsanov M (2018) Differential response of hippocampal and prefrontal oscillations to systemic LPS application. Brain Res 1681:64–74. 10.1016/j.brainres.2017.12.036 29294350 PMC5792247

[B49] Marcantonio ER (2017) Delirium in hospitalized older adults. N Engl J Med 377:1456–1466. 10.1056/NEJMcp1605501 29020579 PMC5706782

[B50] Matsuwaki T, Shionoya K, Ihnatko R, Eskilsson A, Kakuta S, Dufour S, Schwaninger M, Waisman A, Müller W, Pinteaux E, Engblom D, Blomqvist A (2017) Involvement of interleukin-1 type 1 receptors in lipopolysaccharide-induced sickness responses. Brain Behav Immun 66:165–176. 10.1016/j.bbi.2017.06.013 28655587

[B51] McNay EC, Gold PE (2001) Age-related differences in hippocampal extracellular fluid glucose concentration during behavioral testing and following systemic glucose administration. J Gerontol A Biol Sci Med Sci 56:B66–B71. 10.1093/gerona/56.2.b66 11213269

[B52] Murray CL, Skelly DT, Cunningham C (2011) Exacerbation of CNS inflammation and neurodegeneration by systemic LPS treatment is independent of circulating IL-1β and IL-6. J Neuroinflammation 8:50. 10.1186/1742-2094-8-50 21586125 PMC3119173

[B53] Murray C, Sanderson DJ, Barkus C, Deacon RM, Rawlins JN, Bannerman DM, Cunningham C (2012) Systemic inflammation induces acute working memory deficits in the primed brain: relevance for delirium. Neurobiol Aging 33:603–616.e3. 10.1016/j.neurobiolaging.2010.04.002 20471138 PMC3200140

[B54] Murray CL, Obiang P, Bannerman D, Cunningham C (2013) Endogenous IL-1 in cognitive function and anxiety: a study in IL-1RI-/- mice. PLoS One 8:e78385. 10.1371/journal.pone.0078385 24205219 PMC3813582

[B55] Nakamura S, Osaka H, Muramatsu SI, Takino N, Ito M, Aoki S, Jimbo EF, Shimazaki K, Onaka T, Ohtsuki S, Terasaki T, Yamagata T (2017) Gene therapy for a mouse model of glucose transporter-1 deficiency syndrome. Mol Genet Metab Rep 10:67–74. 10.1016/j.ymgmr.2016.12.008 28119822 PMC5238605

[B56] Oguri S, Motegi K, Iwakura Y, Endo Y (2002) Primary role of interleukin-1 alpha and interleukin-1 beta in lipopolysaccharide-induced hypoglycemia in mice. Clin Diagn Lab Immunol 9:1307–1312. 10.1128/cdli.9.6.1307-1312.2002 12414765 PMC130127

[B57] Park MJ, Guest CB, Barnes MB, Martin J, Ahmad U, York JM, Freund GG (2008) Blocking of beta-2 adrenergic receptors hastens recovery from hypoglycemia-associated social withdrawal. Psychoneuroendocrinology 33:1411–1418. 10.1016/j.psyneuen.2008.08.005 18814973 PMC2636565

[B58] Park MJ, Yoo SW, Choe BS, Dantzer R, Freund GG (2012) Acute hypoglycemia causes depressive-like behaviors in mice. Metab Clin Exp 61:229–236. 10.1016/j.metabol.2011.06.013 21820138 PMC3245368

[B59] Ryan DJ, O'Regan NA, Caoimh RÓ, Clare J, O'Connor M, Leonard M, McFarland J, Tighe S, O'Sullivan K, Trzepacz PT, Meagher D, Timmons S (2013) Delirium in an adult acute hospital population: predictors, prevalence and detection. BMJ Open 3:e001772 10.1136/bmjopen-2012-001772 PMC3549230 23299110

[B60] Sanchez JJ, Bidot CJ, O'Phelan K, Gajavelli S, Yokobori S, Olvey S, Jagid J, Garcia JA, Nemeth Z, Bullock R (2013) Neuromonitoring with microdialysis in severe traumatic brain injury patients. Acta Neurochir Suppl 118:223–227. 23564137 10.1007/978-3-7091-1434-6_42

[B61] Saper CB, Romanovsky AA, Scammell TE (2012) Neural circuitry engaged by prostaglandins during the sickness syndrome. Nat Neurosci 15:1088–1095. 10.1038/nn.3159 22837039 PMC3748818

[B62] Schedlowski M, Engler H, Grigoleit JS (2014) Endotoxin-induced experimental systemic inflammation in humans: a model to disentangle immune-to-brain communication. Brain Behav Immun 35:1–8. 10.1016/j.bbi.2013.09.015 24491305

[B63] Schreuder L, Eggen BJ, Biber K, Schoemaker RG, Laman JD, de Rooij SE (2017) Pathophysiological and behavioral effects of systemic inflammation in aged and diseased rodents with relevance to delirium: a systematic review. Brain Behav Immun 62:362–381. 10.1016/j.bbi.2017.01.010 28088641

[B64] Semmler A, Hermann S, Mormann F, Weberpals M, Paxian SA, Okulla T, Schäfers M, Kummer MP, Klockgether T, Heneka MT (2008) Sepsis causes neuroinflammation and concomitant decrease of cerebral metabolism. J Neuroinflammation 5:38. 10.1186/1742-2094-5-38 18793399 PMC2553764

[B65] Shehab N, Lovegrove MC, Geller AI, Rose KO, Weidle NJ, Budnitz DS (2016) US emergency department visits for outpatient adverse drug events, 2013-2014. JAMA 316:2115–2125. 10.1001/jama.2016.16201 27893129 PMC6490178

[B66] Skelly DT, Hennessy E, Dansereau MA, Cunningham C (2013) A systematic analysis of the peripheral and CNS effects of systemic LPS, IL-1β, [corrected] TNF-α and IL-6 challenges in C57BL/6 mice. PLoS One 8:e69123. 10.1371/journal.pone.0069123 23840908 PMC3698075

[B67] Skelly DT, Griffin É, W, Murray CL, Harney S, O'Boyle C, Hennessy E, Dansereau M-A, Nazmi A, Tortorelli L, Rawlins JN, Bannerman DM, Cunningham C (2019) Acute transient cognitive dysfunction and acute brain injury induced by systemic inflammation occur by dissociable IL-1-dependent mechanisms. Mol Psychiatry 24:1533–1548. 10.1038/s41380-018-0075-8 29875474 PMC6510649

[B68] Sonneville R, de Montmollin E, Poujade J, Garrouste-Orgeas M, Souweine B, Darmon M, Mariotte E, Argaud L, Barbier F, Goldgran-Toledano D, Marcotte G, Dumenil AS, Jamali S, Lacave G, Ruckly S, Mourvillier B, Timsit JF (2017) Potentially modifiable factors contributing to sepsis-associated encephalopathy. Intensive Care Med 43:1075–1084. 10.1007/s00134-017-4807-z 28466149

[B69] Stone EA, Lehmann ML, Lin Y, Quartermain D (2006) Depressive behavior in mice due to immune stimulation is accompanied by reduced neural activity in brain regions involved in positively motivated behavior. Biol Psychiatry 60:803–811. 10.1016/j.biopsych.2006.04.020 16814258

[B70] Tahir M, Malik SS, Ahmed U, Kozdryk J, Naqvi SH, Malik A (2018) Risk factors for onset of delirium after neck of femur fracture surgery: a prospective observational study. SICOT J 4:27. 10.1051/sicotj/2018018 29995625 PMC6040837

[B71] Tang M, Gao G, Rueda CB, Yu H, Thibodeaux DN, Awano T, Engelstad KM, Sanchez-Quintero MJ, Yang H, Li F, Li H, Su Q, Shetler KE, Jones L, Seo R, McConathy J, Hillman EM, Noebels JL, De Vivo DC, Monani UR (2017) Brain microvasculature defects and Glut1 deficiency syndrome averted by early repletion of the glucose transporter-1 protein. Nat Commun 8:14152. 10.1038/ncomms14152 28106060 PMC5263887

[B72] Tannahill GM, Curtis AM, Adamik J, Palsson-McDermott EM, McGettrick AF, Goel G, Frezza C, Bernard NJ, Kelly B, Foley NH, Zheng L, Gardet A, Tong Z, Jany SS, Corr SC, Haneklaus M, Caffrey BE, Pierce K, Walmsley S, Beasley FC, et al (2013) Succinate is an inflammatory signal that induces IL-1β through HIF-1α. Nature 496:238–242. 10.1038/nature11986 23535595 PMC4031686

[B73] Teeling JL, Felton LM, Deacon RM, Cunningham C, Rawlins JN, Perry VH (2007) Sub-pyrogenic systemic inflammation impacts on brain and behavior, independent of cytokines. Brain Behav Immun 21:836–850. 10.1016/j.bbi.2007.01.012 17367989

[B74] Thorell A, Efendic S, Gutniak M, Häggmark T, Ljungqvist O (1994) Insulin resistance after abdominal surgery. Br J Surg 81:59–63. 10.1002/bjs.1800810120 8313123

[B75] Tooke BP, Yu H, Adams JM, Jones GL, Sutton-Kennedy T, Mundada L, Qi NR, Low MJ, Chhabra KH (2019) Hypothalamic POMC or MC4R deficiency impairs counterregulatory responses to hypoglycemia in mice. Mol Metab 20:194–204. 10.1016/j.molmet.2018.11.004 30503832 PMC6358536

[B76] Virkamäki A, Puhakainen I, Koivisto VA, Vuorinen-Markkola H, Yki-Järvinen H (1992) Mechanisms of hepatic and peripheral insulin resistance during acute infections in humans. J Clin Endocrinol Metab 74:673–679. 10.1210/jcem.74.3.1740504 1740504

[B77] Voss M, Lorenz NI, Luger AL, Steinbach JP, Rieger J, Ronellenfitsch MW (2018) Rescue of 2-deoxyglucose side effects by ketogenic diet. Int J Mol Sci 19:2462 10.3390/ijms19082462 PMC6121440 30127309

[B78] Wang A, Huen SC, Luan HH, Yu S, Zhang C, Gallezot JD, Booth CJ, Medzhitov R (2016) Opposing effects of fasting metabolism on tissue tolerance in bacterial and viral inflammation. Cell 166:1512–1525.e12. 10.1016/j.cell.2016.07.026 27610573 PMC5555589

[B79] Watne LO, Hall RJ, Molden E, Raeder J, Frihagen F, MacLullich AM, Juliebø V, Nyman A, Meagher D, Wyller TB (2014a) Anticholinergic activity in cerebrospinal fluid and serum in individuals with hip fracture with and without delirium. J Am Geriatr Soc 62:94–102. 10.1111/jgs.12612 24383557

[B80] Watne LO, Torbergsen AC, Conroy S, Engedal K, Frihagen F, Hjorthaug GA, Juliebo V, Raeder J, Saltvedt I, Skovlund E, Wyller TB (2014b) The effect of a pre- and postoperative orthogeriatric service on cognitive function in patients with hip fracture: randomized controlled trial (Oslo Orthogeriatric Trial). BMC Med 12:63. 10.1186/1741-7015-12-63 24735588 PMC4022270

[B81] Yirmiya R, Goshen I (2011) Immune modulation of learning, memory, neural plasticity and neurogenesis. Brain Behav Immun 25:181–213. 10.1016/j.bbi.2010.10.015 20970492

[B82] Zhang WM, Natowicz MR (2013) Cerebrospinal fluid lactate and pyruvate concentrations and their ratio. Clin Biochem 46:694–697. 10.1016/j.clinbiochem.2012.11.008 23195138

